# Deubiquitinase OTUD5 as a Novel Protector against 4‐HNE‐Triggered Ferroptosis in Myocardial Ischemia/Reperfusion Injury

**DOI:** 10.1002/advs.202301852

**Published:** 2023-08-08

**Authors:** Lulu Liu, Jiaojiao Pang, Dandan Qin, Ruochuan Li, Dan Zou, Kai Chi, Wenxiao Wu, Haiying Rui, Huaxiang Yu, Wenyong Zhu, Kai Liu, Xuting Wu, Jinxin Wang, Ping Xu, Xiaoshuai Song, Yihai Cao, Jiali Wang, Feng Xu, Li Xue, Yuguo Chen

**Affiliations:** ^1^ Department of Emergency Medicine Qilu Hospital of Shandong University Jinan 250012 China; ^2^ Shandong Provincial Clinical Research Center for Emergency and Critical Care Medicine Institute of Emergency and Critical Care Medicine of Shandong University Chest Pain Center Qilu Hospital of Shandong University Jinan 250012 China; ^3^ Key Laboratory of Emergency and Critical Care Medicine of Shandong Province Key Laboratory of Cardiopulmonary‐Cerebral Resuscitation Research of Shandong Province Shandong Provincial Engineering Laboratory for Emergency and Critical Care Medicine Qilu Hospital of Shandong University Jinan 250012 China; ^4^ Shandong Key Laboratory Magnetic Field‐free Medicine & Functional Imaging (MF) Qilu Hospital of Shandong University Jinan 250012 China; ^5^ NMPA Key Laboratory for Clinical Research and Evaluation of Innovative Drug Qilu Hospital of Shandong University Jinan 250012 China; ^6^ Department of Thoracic Surgery Qilu Hospital of Shandong University Qingdao 266035 China; ^7^ Department of Cardiovascular surgery Qilu Hospital of Shandong University Jinan 250012 China; ^8^ Department of Microbiology Tumor and Cell Biology Karolinska Institute Stockholm 171 65 Sweden

**Keywords:** 4‐hydroxy‐2‐nonenal (4‐HNE), ferroptosis, glutathione peroxidase 4 (GPX4), myocardial ischemia reperfusion injury, ovarian tumor (OTU) deubiquitinase 5 (OTUD5)

## Abstract

Despite the development of advanced technologies for interventional coronary reperfusion after myocardial infarction, a substantial number of patients experience high mortality due to myocardial ischemia‐reperfusion (MI/R) injury. An in‐depth understanding of the mechanisms underlying MI/R injury can provide crucial strategies for mitigating myocardial damage and improving patient survival. Here, it is discovered that the 4‐hydroxy‐2‐nonenal (4‐HNE) accumulates during MI/R, accompanied by high rates of myocardial ferroptosis. The loss‐of‐function of aldehyde dehydrogenase 2 (ALDH2), which dissipates 4‐HNE, aggravates myocardial ferroptosis, whereas the activation of ALDH2 mitigates ferroptosis. Mechanistically, 4‐HNE targets glutathione peroxidase 4 (GPX4) for K48‐linked polyubiquitin‐related degradation, which 4‐HNE‐GPX4 axis commits to myocyte ferroptosis and forms a positive feedback circuit. 4‐HNE blocks the interaction between GPX4 and ovarian tumor (OTU) deubiquitinase 5 (OTUD5) by directly carbonylating their cysteine residues at C93 of GPX4 and C247 of OTUD5, identifying OTUD5 as the novel deubiquitinase for GPX4. Consequently, the elevation of OTUD5 deubiquitinates and stabilizes GPX4 to reverse 4‐HNE‐induced ferroptosis and alleviate MI/R injury. The data unravel the mechanism of 4‐HNE in GPX4‐dependent ferroptosis and identify OTUD5 as a novel therapeutic target for the treatment of MI/R injury.

## Graphical abstract

4‐hydroxy‐2‐nonenal (4‐HNE), accumulated in myocardial ischemia/reperfusion, induces ferroptosis, and aggravates myocardial injury. In cardiomyocytes, 4‐HNE blocks the interaction between glutathione peroxidase 4 (GPX4) and ovarian tumor (OTU) deubiquitinase 5 (OTUD5) by addition and carbonylation, which promotes GPX4 ubiquitination and degradation. These findings unravel the mechanism of 4‐HNE in GPX4‐dependent ferroptosis and identify OTUD5 as a novel therapeutic target for the treatment of myocardial ischemia/reperfusion injury.

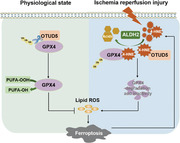

## 1 Introduction

Ischemic heart disease remains the leading cause of morbidity and mortality around the world.^[^ ^1^, ^2^ ^]^ Although timely interventional coronary reperfusion effectively salvages the ischemic myocardium, a substantial number of patients are still experiencing high mortality due to myocardial ischemia‐reperfusion (MI/R) injury.^[^ ^3^ ^]^ Exploring the mechanisms underlying MI/R would provide crucial strategies for mitigating myocardial damage and improving patient survival. Programmed cardiomyocyte death has been identified as an important mechanism involved in the initiation and development of MI/R injury.^[^ ^4^ ^]^ Therefore, deepening understanding of cardiomyocyte death to identify novel therapeutic targets is still a huge medical need.

Ferroptosis is a new type of regulated cell death driven by excessive iron‐dependent lipid peroxides.^[^ ^5^, ^6^ ^]^ Accumulating evidence has shown that ferroptosis plays a key role in the pathogenesis and progression of diverse cardiovascular diseases, including MI/R.^[^ ^7^ ^]^ Targeting ferroptosis may be a potential strategy for the management of MI/R injury.^[^ ^8^ ^]^ Glutathione Peroxidase 4 (GPX4), which converts lipid peroxides to less harmful lipid alcohols, has emerged as a key regulator that inhibits ferroptosis. It has been reported that MI/R‐induced ferroptosis is accompanied by inhibition of GPX4, whereas upregulation of GPX4 attenuates myocardial damage and improves cardiac function.^[^ ^9^, ^10^ ^]^ Until recently, research has shown that ubiquitin–proteasome degradation regulates GPX4 stability.^[^ ^11^, ^12^ ^]^ Thus, identifying regulators of GPX4 may be a promising therapeutic target in ferroptosis during MI/R.

4‐hydroxy‐2‐nonenal (4‐HNE) is a well‐known by‐product of lipid peroxidation and an important marker of ferroptosis. It is a highly reactive and toxic aldehyde. Higher 4‐HNE concentrations (above 10–20 µmol L^−1^) can be detrimental to cell survival.^[^ ^13^, ^14^ ^]^ It can form adducts with macromolecules, including DNA, lipids, and proteins, resulting in their carbonylation and disrupting their structure and function. The accumulation of 4‐HNE has been detected in various cardiovascular diseases, which increases the carbonylation of proteins, including sirtuin 1 (SIRT1),^[^ ^15^ ^]^ voltage‐dependent anion channel1 (VDAC1), mitoCa^2+^ uniporter (MCU),^[^ ^16^ ^]^ and succinate dehydrogenase (SDH),^[^ ^17^ ^]^ impairs cellular homeostasis, and promotes cardiac dysfunction. Furthermore, our earlier studies and others have verified that clearance of 4‐HNE is beneficial for alleviating MI/R injury.^[^ ^18^, ^19^, ^20^ ^]^ In particular, we found that 4‐HNE could provoke cardiomyocyte death, including cellular apoptosis and necroptosis.^[^ ^20^ ^]^ However, whether 4‐HNE exerts regulatory effects on ferroptosis to form a positive feedback circuit, resulting in an amplification cascade of myocyte damage, remains unknown.

The subfamily of ovarian tumor (OTU) deubiquitinases have been the focus in many essential cellular processes. Recent progress in elucidating the functions of ovarian tumor (OTU) deubiquitinase 5(OTUD5), the member of OTU family, has greatly enhanced our understanding in this area. OTUD5 negatively regulate IFN‐I expression by downregulating TRAF3 K63‐linked polyubiquitination.^[^ ^21^ ^]^ In addition, OTUD5 promotes DNA damage repair by inhibiting Ku80 degradation or regulating FACT‐dependent transcription at damaged chromatin.^[^ ^22^, ^23^ ^]^ Meanwhile, OTUD5 can regulate tumor and immunity by hydrolyzing specific linkage types within polyubiquitin to modulate substrates stability or activity.^[^ ^24^, ^25^ ^]^ However, there are few reports addressing the function of OTUD5 in cardiovascular disease.

In the present study, we elucidated the feedback regulation of 4‐HNE on cardiomyocyte ferroptosis during MI/R injury. 4‐HNE can directly evoke myocyte ferroptosis by interacting with GPX4 and the deubiquitinase OTUD5 at cysteine residues, leading to increased ubiquitination and degradation of GPX4. Thus, our findings provide new insights into the pathogenesis of ferroptosis and identify OTUD5 as a potential novel therapeutic target for preventing MI/R injury.

## 2 Results

### 2.1 MI/R‐Induced 4‐HNE Accumulation and Ferroptosis are Aggravated in ALDH2‐Deficient Mice

It is well established that ferroptosis plays an important role in MI/R injury.^[^ ^26^, ^27^ ^]^ The MI/R model was established in mice by left anterior descending coronary artery (LAD) ligation for 30 min of ischemia followed by 24 h of reperfusion. Interestingly, cardiac ferroptosis in MI/R was identified using RNA‐sequencing (RNA‐seq) (Figure S1A, Supporting Information). In addition, ferroptosis and protein degradation pathways were significantly enriched in myocardial I/R injury through enrichment analysis. Using a comprehensive characterization strategy with high sensitivity and facility for fatty aldehydes based on derivatization and high‐performance liquid chromatography‐multiple reaction monitoring (HPLC‐MRM),^[^ ^28^ ^]^ 4‐HNE was significantly increased in the hearts subjected to MI/R (Figure S1B, Supporting Information). As shown in Figure S1C (Supporting Information), we further verified that 4‐HNE levels increased with time after MI/R, accompanied by the occurrence of ferroptosis.

Ferroptosis is driven by iron‐dependent lipid peroxides, and 4‐HNE, a reactive lipid peroxidation product, serves as a biomarker of ferroptosis. Aldehyde dehydrogenase 2 (ALDH2) is the main enzyme that metabolizes 4‐HNE.^[^ ^15^, ^19^, ^20^ ^]^ We analyzed cell death pathways in GSE59100, which is a study on human induced pluripotent stem cell‐derived cardiomyocytes generated from individuals carrying the most common heterozygous form of the ALDH2*2 genotype. We found that ferroptosis was more significant in the ischemic model of the ALDH2*2 genotype than in the WT, and played an important role in all cell death pathways (Figure S1D, Supporting Information).

To elucidate whether 4‐HNE affects feedback circuitries to ferroptosis during MI/R, we generated cardiomyocyte‐specific knockout mice of ALDH2 (Figure S2A, Supporting Information). The protein expression level of ALDH2 was examined using western blotting (Figure S2B, Supporting Information). Under basal conditions, there were no significant alterations in heart size (Figure S2C, Supporting Information), heart weight (Figure S2D, Supporting Information), or cardiac function (Table S1, Supporting Information) in ALDH2 cKO mice compared to control animals.

**Figure 1:**
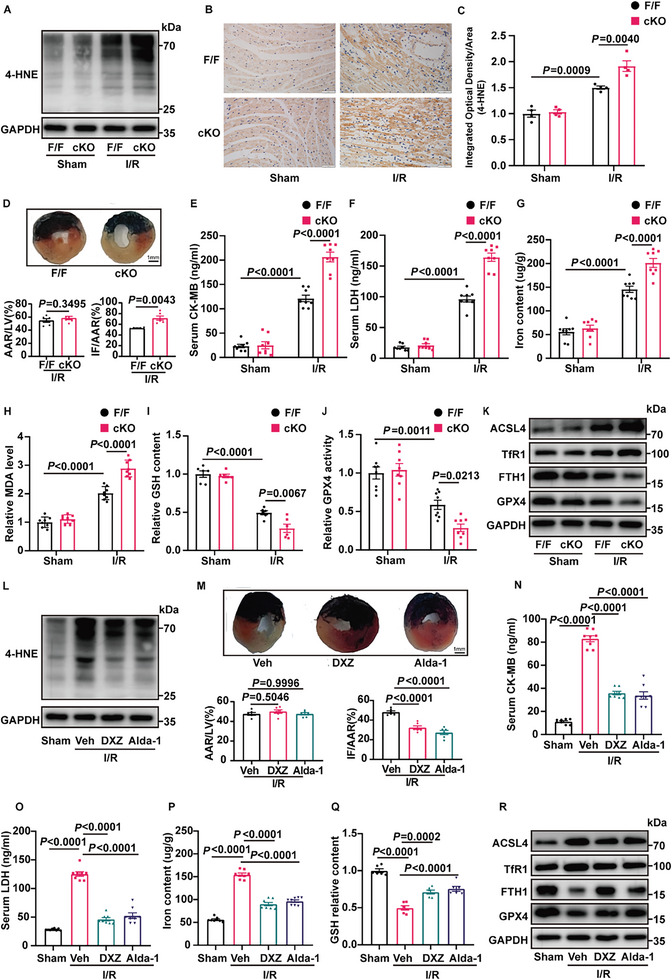
ALDH2 regulates 4‐HNE accumulation and ferroptosis in MI/R. A) Protein expression of 4‐HNE in ALDH2^flox/flox^ (F/F) and cardiomyocyte‐specific ALDH2‐knockout (cKO) mice hearts subjected to MI/R (30‐min ischemia/24‐h reperfusion, n = 4). B,C) Representative immunohistochemical staining results indicating 4‐HNE expression in F/F and cKO mice heart tissues 24 h after MI/R surgery (n = 4). Scale bar:50 µm. D) Representative photographs and statistical analysis of infarct size (IF) and area at risk (AAR) in mice hearts stained with Evans blue dye (EBD) and triphenyl tetrazolium chloride (TTC) (n = 6). The hearts were collected 24 h after MI/R surgery. Scale bar: 1 mm. E,F) Serum levels of CK‐MB and LDH in F/F and cKO mice with sham or MI/R injury (30‐min ischemia/24‐h reperfusion, n = 8). G‐J) Iron content, MDA, GSH, and GPX4 activity in F/F and cKO mice 24 h after MI/R surgery (n = 6–8). K) Western blotting of the ferroptosis marker protein (ACSL4, TfR1, FTH1, and GPX4) in F/F and cKO mice hearts subjected to MI/R (30‐min ischemia/24‐h reperfusion, n = 4). L) Representative western blotting of 4‐HNE in WT mice subjected to MI/R (30‐min ischemia/24‐h reperfusion). The mice were treated with DXZ (50 mg/kg), Alda‐1 (25 mg kg^−1^) or vehicle (solvent control) (n = 4). M) EBD/TTC staining of heart sections collected from the DXZ (50 mg kg^−1^), Alda‐1 (25 mg kg^−1^), and vehicle groups(n = 6). The hearts were collected 24 hours after MI/R surgery, and the infarct area and at‐risk area were evaluated. Scale bar: 1 mm. N,O) Serum levels of CK‐MB and LDH in mice treated with DXZ (50 mg kg^−1^), Alda‐1 (25 mg kg^−1^) and vehicle 24 h after MI/R surgery (n = 8). P,Q) Iron content, and GSH levels in WT mice with DXZ (50 mg/kg) or Alda‐1 (25 mg/kg) subjected to MI/R (30‐min ischemia/24‐h reperfusion, n = 6–8). R) Protein levels of ACSL4, TfR1, FTH1, and GPX4 in WT mice heart tissues from DXZ (50 mg kg^−1^), Alda‐1 (25 mg kg^−1^) and vehicle groups. The hearts were collected from 24 hours after MI/R surgery (n = 4). Data are expressed as mean ± SEM. Unpaired two‐tailed Student's t‐test (analysis in AAR/LV) and Mann‐Whitney test (analysis in IF/AAR) were used for the analysis in (D). One‐way analysis of variance (ANOVA) was used for the analysis in E–J,M–Q).

We examined 4‐HNE accumulation and myocardial injury in ALDH2 cKO mice subjected to MI/R. 4‐HNE was further increased in I/R‐injured hearts from the ALDH2 cKO group (**Figure** **1**A–C). As expected, ALDH2 cKO mice showed increased susceptibility to MI/R injury, represented by enlarged myocardial infarct size, enhanced creatine kinase MB (CK‐MB), and lactate dehydrogenase (LDH) levels (Figure 1D–F). To determine whether the effect of ALDH2 is associated with ferroptosis in MI/R, we evaluated several parameters of ferroptosis. Indeed, MI/R‐induced ferroptosis was aggravated by ALDH2 deficiency, manifesting as iron accumulation, malondialdehyde (MDA) production, glutathione (GSH) depletion, and GPX4 inactivation (Figure 1G–J). Consistently, the same results were confirmed by western blotting of ferroptosis‐related proteins (Figure 1K). ALDH2 knockout promoted the expression of acyl‐CoA synthetase long‐chain family member 4 (ACSL4), transferrin receptor 1 (TfR1) and downregulated the expression of ferritin heavy chain 1 (FTH1), GPX4 in I/R‐induced cardiac tissues.

### 2.2 MI/R‐Induced 4‐HNE Accumulation and Ferroptosis are Alleviated by ALDH2 Activation

We further enhanced the ALDH2 activity to elucidate the role of 4‐HNE in ferroptosis. Mice were pretreated with either the iron chelator dexrazoxane (DXZ) or the ALDH2 activator Alda‐1, and then subjected to I/R. Our results revealed that pretreatment with both DXZ and Alda‐1 significantly decreased 4‐HNE accumulation (Figure 1L). Importantly, MI/R‐increased infarct size, CK‐MB, and LDH levels were reduced in the presence of DXZ or Alda‐1 (Figure 1M–O). As shown in Figure 1P,Q, both DXZ and Alda‐1 administration prevented the I/R‐induced increase in Fe^2+^ and reduction of GSH. Moreover, the expression of ACSL4 and TfR1 was upregulated, and the levels of FTH1 and GPX4 were downregulated in the MI/R group, which were restored by incubation with DXZ or Alda‐1 (Figure 1R).

H9c2 cells were exposed to hypoxia followed by reoxygenation (H/R) to mimic MI/R in vitro. H/R exposure inhibited myocardial cell viability, and the inhibitor of ferroptosis and activator of ALDH2 significantly reduced H/R‐induced cell death (Figure S3A, Supporting Information). In line with the in vivo results, both DXZ and Alda‐1 pretreatment restrained ferroptosis in H/R‐treated H9c2 cells, expressed as a decrease in iron content, MDA, and ROS production (Figure S3B–E, Supporting Information). Taken together, these results suggest that ALDH2 activation has similar effects as blocking ferroptosis, indicating that 4‐HNE might be a regulator of ferroptosis in MI/R.

### 2.3 4‐HNE Provokes Ferroptosis and Reduces GPX4 Expression In Vitro

**Figure 2:**
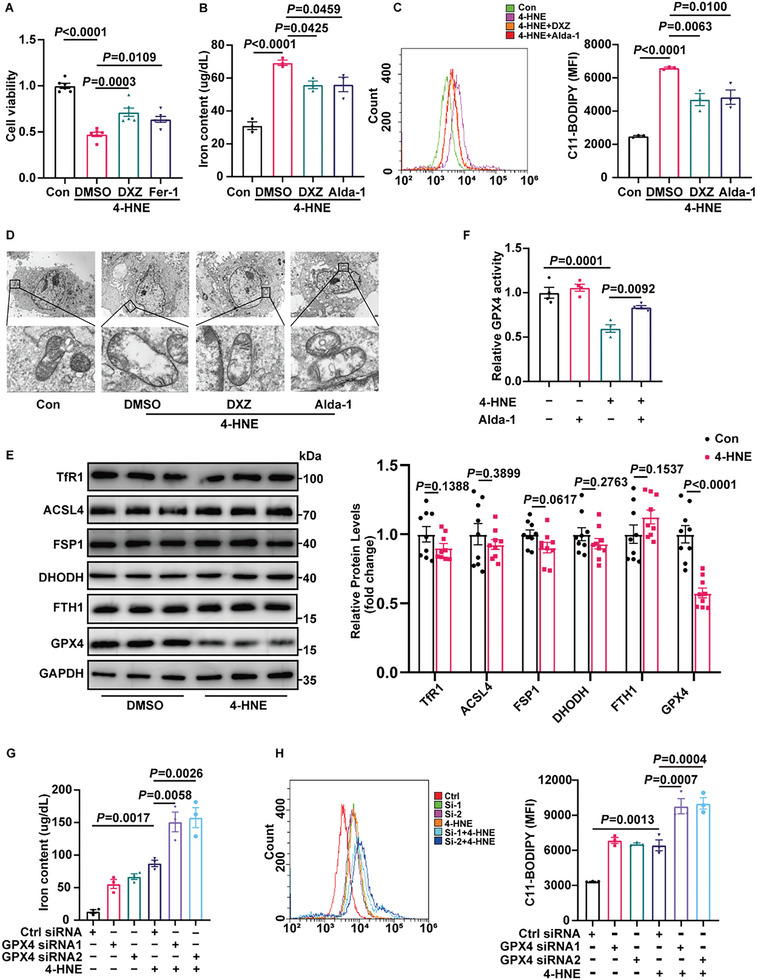
4‐HNE reduces GPX4 expression and induces cardiomyocyte ferroptosis. A) Cell viability was measured in H9c2 cells treated with 4‐HNE (40 µM) for 6 h in the presence of DXZ (10 µM), Fer‐1 (1 µM) or DMSO (n = 6). B,C) Iron content and lipid ROS of H9c2 cells under the indicated treatments (n = 3). D) Representative images of mitochondrial injury in H9c2 cells observed using transmission electron micrographs (n = 3). Scale bars:10 µm (top) and 1 µm (bottom). E) Western blotting analysis of the ferroptosis proteins under 4‐HNE treatment (40 µM,6 h) in NRCMs (n = 9). F, The activity of GPX4 under 4‐HNE (40 µM) treatment in the presence or absence of Alda‐1 (20 µM) for 6 h (n = 4). G,H) Iron content and lipid ROS in NRCMs treated with 4‐HNE (40 µM,6 h) after transfection with control siRNA (NC) or two siRNAs targeting GPX4 (n = 3). Data are expressed as mean ± SEM. Unpaired two‐tailed Student's t‐test was used for the analysis in (E). One‐way ANOVA was used for the analysis in A–C,F–H).

Previous studies have reported that high levels of 4‐HNE are toxic to the myocardial cells. To investigate the role of ferroptosis in 4‐HNE‐induced cardiotoxicity, we used the CCK‐8 assay to measure cell viability in the presence of DXZ and Ferrostatin‐1 (Fer‐1). Our results revealed that ferroptosis inhibitors had protective effects against 4‐HNE‐induced cell death (**Figure** **2**A).

As ferroptosis is characterized by iron overload and lipid peroxide accumulation, we quantified labile Fe^2+^ and lipid peroxidation in cardiomyocytes to identify the role of 4‐HNE in ferroptosis. As displayed in Figure 2B,C, H9c2 cells exhibited a remarkable increase in iron content and lipid ROS level in the 4‐HNE group, which were reduced by the administration of DXZ and Alda‐1. Transmission electron microscopy (TEM) revealed that 4‐HNE incubation with cells caused vacuolization of mitochondria and rupture of the outer mitochondrial membrane, which are characteristic changes in ferroptosis. In addition, DXZ and Alda‐1 attenuated the changes in mitochondria (Figure 2D). These results indicate that 4‐HNE promotes ferroptosis and that inhibition of ferroptosis decreases the cytotoxicity of 4‐HNE.

We assessed the expression levels of key players associated with ferroptosis. Among them, GPX4, the pivotal molecule in ferroptosis, was reduced upon 4‐HNE incubation (Figure 2E). As observed in Figure S4A (Supporting Information), remarkably decreased protein expression of GPX4 was detected in neonatal rat cardiomyocytes (NRCM) with 40 µM 4‐HNE treatment for 6 h. The immunofluorescence assay of GPX4 showed similar results in 4‐HNE treated H9c2 cells (Figure S4B, Supporting Information). The level of GPX4 was significantly reversed by DXZ or Alda‐1 treatment (Figure S4C, Supporting Information). Moreover, the activity of GPX4 was also reduced upon 4‐HNE stimulation (Figure 2F). These data suggest that GPX4 disruption contributes to 4‐HNE‐induced ferroptosis.

To verify whether the effect of 4‐HNE on ferroptosis was due to GPX4 downregulation, NRCMs were transfected with GPX4 siRNAs (Figure S4D, Supporting Information) and then treated with 4‐HNE. As determined by iron content and lipid ROS levels, 4‐HNE‐induced ferroptosis was aggravated by GPX4 knockdown (Figure 2G,H). In addition, we used Crispr‐cas9 to knockout GPX4 and constructed the H9c2 stable cell line with lentivirus. We performed iron content and lipid ROS assays in GPX4 control and KO cells, and found that 4‐HNE‐induced ferroptosis was aggravated by knocking out GPX4 (Figure S4E–G, Supporting Information). Collectively, our results suggest that the cytotoxicity of 4‐HNE is associated with GPX4 dysfunction‐mediated ferroptosis.

### 2.4 4‐HNE Promotes Ubiquitin–Proteasomal Degradation of GPX4

**Figure 3:**
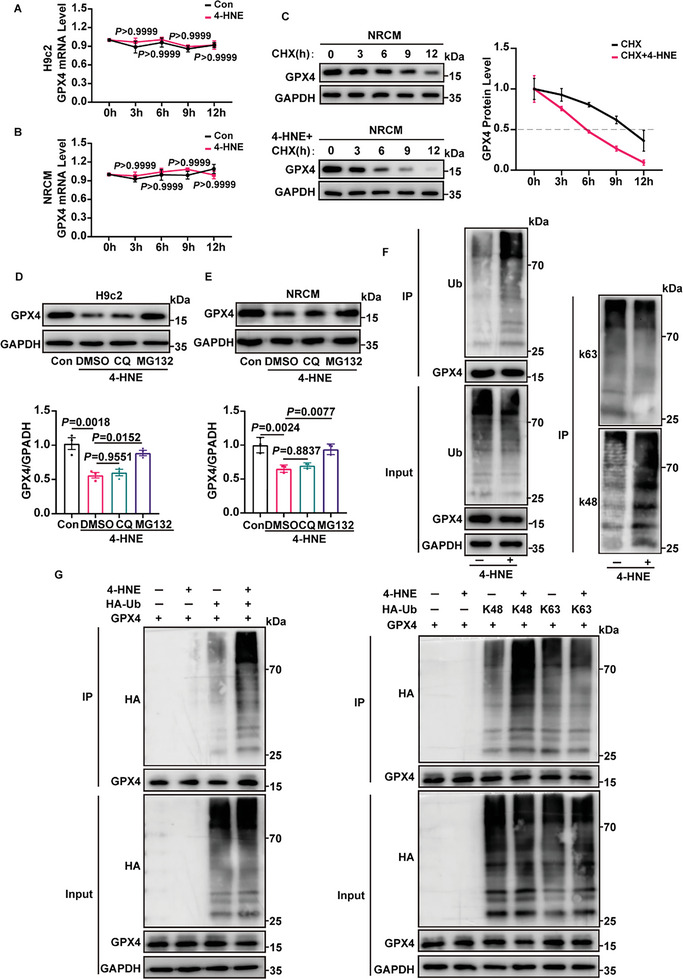
4‐HNE promotes the degradation of GPX4. A,B) The mRNA levels of GPX4 in 4‐HNE (40 µM) treated H9c2 cells and NRCMs at the indicated time points were detected by RT‐qPCR (n = 4). C) The protein levels of GPX4 in cycloheximide (CHX, 10 µM) treated NRCMs at the indicated time points with or without 4‐HNE (40 µM) incubation were detected by western blotting (n = 3). D,E) MG132 (10 µM) or chloroquine (CQ, 10 µM) were preincubated for 30 min, and H9c2 cells and NRCMs were treated with 4‐HNE (40 µM,6 h). Protein levels of GPX4 were analyzed by western blotting (n = 3). F) Representative images showed the total, K48‐, and K63‐linked ubiquitylation by western blotting in immunoprecipitation (IP) assays using anti‐GPX4 antibody (n = 3). G) GPX4, HA‐ubiquitin (HA‐Ub), HA‐K48, and HA‐K63 were transfected into HEK293T cells treated with or without 4‐HNE (40 µM,6 h), and IP assays were performed (n = 3). Data are expressed as mean ± SEM. Two‐way ANOVA was used for the analysis in A,B). One‐way ANOVA was used for the analysis in D,E).

Suppression of GPX4 plays an important role in cardiomyocyte ferroptosis. Since we found that the protein expression of GPX4 was reduced after 4‐HNE treatment, we next determined whether the reduction in GPX4 was regulated at the transcriptional or post‐transcriptional level. Our results revealed that the time course of GPX4 mRNA level was unaffected in 4‐HNE‐treated H9c2 cells or NRCMs (**Figure** **3**A,B).

To verify the regulation of 4‐HNE on GPX4 at the post‐transcriptional level, the protein synthesis inhibitor cycloheximide (CHX) was used to determine the protein level of GPX4 in 4‐HNE treated cells. We found that 4‐HNE treatment shortened the half‐life of GPX4 (Figure 3C). As the ubiquitin‐proteasome system and autophagy‐lysosome pathway are the two main ways for the removal of proteins, we treated cells with either MG132 (a commonly used proteasome inhibitor) or chloroquine (CQ, a lysosome inhibitor) and found that the protein level of GPX4 was sensitive to MG132 but not to CQ (Figure 3D,E). These results indicate that the reduction in GPX4 induced by 4‐HNE is regulated through the proteasome pathway. Consistent with this observation, in NRCMs and HEK293T cells, 4‐HNE treatment increased the ubiquitination of GPX4 on the K48‐linked ubiquitin chain, but not on the K63‐linked ubiquitin chain (Figure 3F,G). Collectively, our data illustrate that 4‐HNE triggers ubiquitin–proteasomal degradation of GPX4 in cardiomyocytes.

### 2.5 OTUD5 Mediates 4‐HNE‐Induced GPX4 Degradation and Ferroptosis

**Figure 4:**
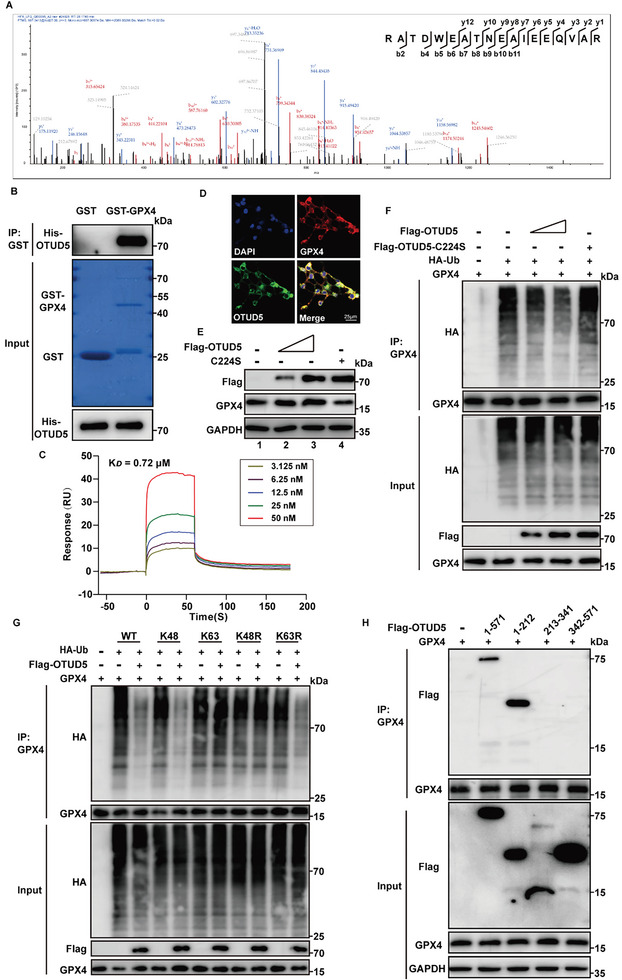
OTUD5 regulates GPX4 stability. A) OTUD5 was identified in the protein mixture enriched by anti‐GPX4 antibody. The graph represented peptide fragment RATDWEATNEAIEEQVAR, which was specifically referred to OTUD5 protein. B) Purified recombinant GST, GST‐GPX4 were incubated with His‐OTUD5 in vitro and the direct interaction between GPX4 and OTUD5 was demonstrated by the GST pull‐down assay (n = 3). C) Surface plasmon resonance (SPR) sensorgrams of the binding of an increasing amount of OTUD5 to GPX4 ligand captured on a CM5 chip. The increase in RUs from baseline was measured and used to calculate the equilibrium dissociation constant (K*_D_*) for OTUD5 binding to immobilized GPX4 ligand. D) Colocalization of GPX4 (red) with OTUD5 (green) was detected in HEK293T cells by immunofluorescence analysis (n = 5). Scale bar:25 µm. E) GPX4 was transfected into HEK293T cells along with increasing amounts of Flag‐OTUD5 or C224S. GPX4 protein levels were detected by western blotting (n = 3). F) GPX4 ubiquitination was analyzed in HEK293T cells transfected with GPX4, HA‐ubiquitin (HA‐Ub), or increasing amounts of Flag‐OTUD5 or C224S (n = 3). G) GPX4, HA‐Ub, or its lysine residue mutants were transfected into HEK293T cells with or without Flag‐OTUD5. Co‐IP assays were performed using anti‐GPX4 antibody (n = 3). H) HEK293T cells transfected with the indicated constructs were subjected to Co‐IP with anti‐GPX4 antibody (n = 3).

Protein ubiquitination is a reversible process controlled by E3 ubiquitin ligases and deubiquitinases (DUBs). We used the database to predict potential ubiquitination‐related enzymes targeting GPX4 (Figure S5A, Supporting Information) and found that E3 ligases MIB2, NEDD4L, STUB1, and deubiquitinase OTUD5 could bind to GPX4 in co‐immunoprecipitation experiments (Figure S5B, Supporting Information). In addition, our results demonstrated that only the binding between OTUD5 and GPX4 was affected under 4‐HNE treatment, while the interactions between E3 ligases (MIB2, NEDD4L, and STUB1) and GPX4 were not changed (Figure S5C, Supporting Information). In addition, the OTUD5 expression was not changed after 4‐HNE treatment (Figure S5D, Supporting Information). Next, we determined the interaction of OTUD5 and GPX4 by co‐immunoprecipitation and mass spectrometry (**Figure** **4**A; Table S2, Supporting Information). The direct interaction of OTUD5 and GPX4 was further confirmed by GST‐pull down, surface plasmon resonance (SPR) and microscale thermophoresis (MST) (Figure 4B,C; Figure S5E, Supporting Information), and the colocalization of OTUD5 and GPX4 proteins in the NRCMs was verified by immunofluorescence (Figure 4D). As OTUD5 belongs to the DUBs family and plays a critical role in controlling the Ub‐dependent degradation of various proteins, 4‐HNE may reduce the interaction between OTUD5 and GPX4 to promote GPX4 ubiquitination and subsequent degradation.

To test whether OTUD5 affects the protein stability of GPX4, we co‐transfected GPX4 with Flag‐OTUD5 or its enzyme‐inactive mutant C224S into HEK293T cells. OTUD5 increased GPX4 protein levels and decreased GPX4 ubiquitination in a dose‐dependent manner, whereas ectopic expression of OTUD5^C224S^ did not affect GPX4 (Figure 4E,F; Figure S5E, Supporting Information), indicating that OTUD5 enzyme activity is indispensable for GPX4 protein stabilization. As shown in ubiquitination assays, OTUD5 significantly downregulated the ubiquitination level of GPX4 in the presence of WT ubiquitin and K48 ubiquitin. Importantly, OTUD5 did not decrease the ubiquitination level of GPX4 in K48R‐transfected cells, but the levels decreased in cells transfected with the ubiquitin mutant K63R, indicating that OTUD5 cleaves the K48‐linked ubiquitination of GPX4 rather than K63‐linked chains (Figure 4G). We then co‐transfected Flag‐tagged truncated OTUD5 mutants with GPX4 and found that the N‐terminal region (1–212 aa) of OTUD5 was required for its interaction with GPX4 (Figure 4H). Taken together, OTUD5 specifically deubiquitinated and stabilized the protein level of GPX4.

**Figure 5:**
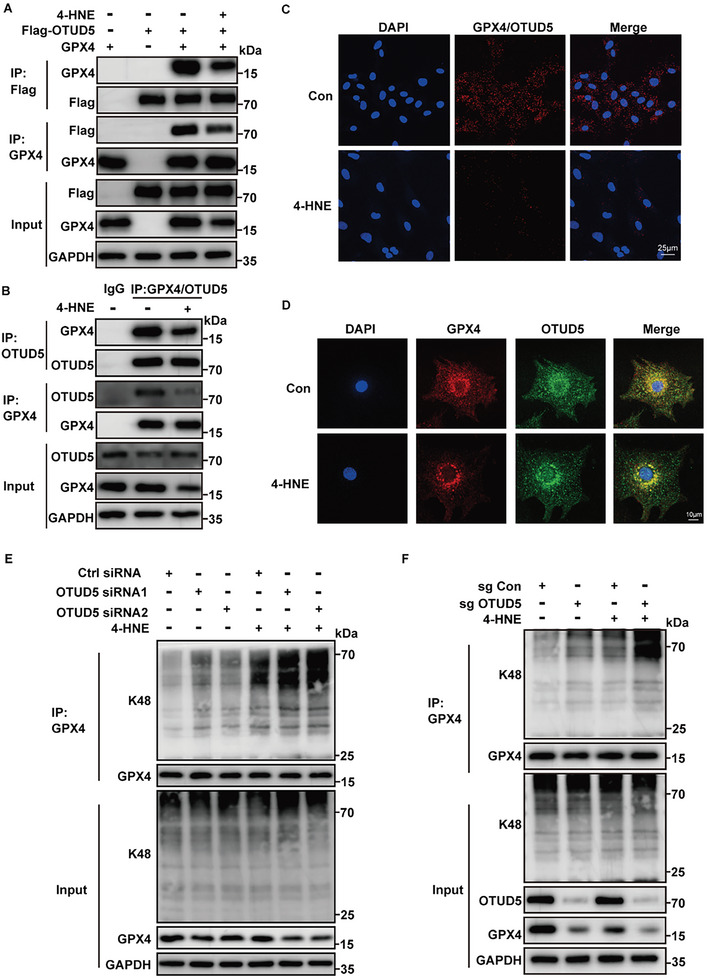
OTUD5 mediates the effects of 4‐HNE on the degradation of GPX4. A) Co‐immunoprecipitation (Co‐IP) was performed with lysates from HEK293T cells transfected with GPX4 and Flag‐OTUD5 plasmids, with or without 4‐HNE treatment (40 µM for 6 h, n = 3). B) Co‐IP was performed on lysates of NRCMs incubated with or without 4‐HNE (40 µM,6 h). Lysates were extracted for Co‐IP with GPX4/OTUD5 specific antibody or control IgG, followed by probing with antibodies specific for GPX4/OTUD5 (n = 4). C) Representative images of proximity ligation assay (red fluorescent dots) between GPX4 and OTUD5 in NRCMs with or without 4‐HNE treatment (40 µM for 6 h, n = 5). Scale bar: 25 µm. D) Colocalization of GPX4 (red) with OTUD5 (green) in NRCMs in the presence or absence of 4‐HNE treatment (40 µM for 6 h, n = 5). Scale bar:10 µm. E) Co‐IP analysis of endogenous GPX4 ubiquitination in NRCMs transfected with control siRNA (NC) or with two siRNAs targeting OTUD5 after 4‐HNE stimulation (40 µM for 6 h, n = 3). F) OTUD5 WT (sg Con) and KO (sg OTUD5) H9c2 cells were treated with 4‐HNE (40 µM,6 h), then GPX4 protein level and the ubiquitination of GPX4 were detected by Immunoblot assays (n = 3).

Furthermore, we confirmed that GPX4 and OTUD5 could form a complex through reciprocal coimmunoprecipitation, immunofluorescence, duolink proximity ligation in situ assay (PLA), and GST‐pull down assay, and that 4‐HNE could weak this binding (**Figure** **5**A–D; Figure S6E, Supporting Information). In addition, OTUD5 rescued the GPX4 de‐ubiquitination phenotype in HEK293T cells, while E3 ligases MIB2, NEDD4L, and STUB1 didn't affect the ubiquitination of GPX4 caused by 4‐HNE (Figure S6A–D, Supporting Information). To investigate whether endogenous OTUD5 contributes to 4‐HNE‐induced degradation of GPX4, we transfected NRCMs with two siRNAs directed against OTUD5. OTUD5 silencing decreased the protein level of GPX4 (Figure S6F, Supporting Information). We also observed that OTUD5 knockdown led to increased GPX4 K48‐linked ubiquitination, and the effect of OTUD5 was more substantial after 4‐HNE treatment (Figure 5E). Similarly, knockout of OTUD5 in H9c2 cells with Crispr‐Cas9 technology also decreased GPX4 protein levels and increased GPX4 K48‐linked ubiquitination under 4‐HNE treatment (Figure 5F; Figure S6G, Supporting Information). Overall, these observations indicated that OTUD5 mediates 4‐HNE‐induced GPX4 downregulation.

To confirm the effects of OTUD5 on ferroptosis more comprehensively, we performed RNA‐seq to characterize transcriptome‐wide changes in OTUD5 deficient NRCMs. A total of 2904 differentially expressed genes had significant changes (q<0.05 and Log_2_FC> = 1). Compared with control cells, 863 genes were up‐regulated and 2041 genes were down‐regulated (Figure S7A, Supporting Information). Among them, ferroptosis related genes were significantly affected (Figure S7B, Supporting Information). Gene Ontology (GO) and Kyoto Encyclopedia of Genes and Genomes (KEGG) analysis identified that OTUD5 silencing was associated with ferroptosis, ubiquitin mediated proteolysis, and cardiomyocyte function (Figure S7C, Supporting Information). Gene Set Enrichment Analysis (GSEA) results showed that OTUD5 silencing was associated with arachidonic acid metabolism and iron ion transport, which are essential factors for ferroptosis (Figure S7D, Supporting Information). Taken together, these results indicate that OTUD5 depletion results in increased ferroptosis in cardiomyocytes.

### 2.6 4‐HNE Affects the Interaction between OTUD5 and GPX4 by Addition and Carbonylation

**Figure 6:**
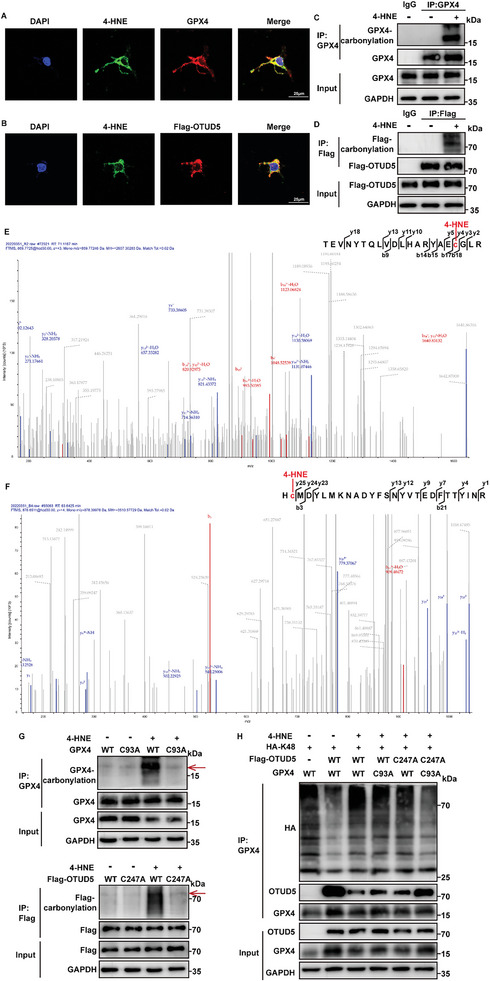
4‐HNE‐induced carbonylation inhibits the interaction between OTUD5 and GPX4. A,B) Staining of 4‐HNE (green), GPX4 or Flag‐OTUD5(red) in HEK293T cells (n = 5). Scale bar: 25 µm. C,D) Immunoblot assays of carbonylation of GPX4 and OTUD5 in HEK293T cells transfected with GPX4 and Flag‐OTUD5 detected by selective labeling with m‐APA (n = 3). E,F) LC‐MS spectra of 4‐HNE modification of GPX4(E) and OTUD5(F) at the Cys residue. G) Immunoblot assays of carbonylation in HEK293T cells transfected with GPX4 WT, GPX4 mutants (C93A) or Flag‐OTUD5 WT, Flag‐OTUD5 mutants (C247A) by oxyblot technology (n = 3). The targeted protein bands are indicated by the red arrows. H) HEK293T cells were co‐transfected with indicated plasmids. Co‐IP assays were performed using anti‐GPX4 antibody and the levels of GPX4 ubiquitination were examined by western blotting (n = 3).

4‐HNE can bind to cysteine, histidine, and lysine amino residues by Michael reactions, which results in the formation of covalent lipid‐protein adducts and protein carbonylation, leading to the inhibition of protein function.^[^ ^29^ ^]^ Based on these, we hypothesized that carbonyl stress may be involved in the decrease of GPX4. Immunofluorescence analysis indicated that 4‐HNE colocalized with GPX4 and OTUD5 (**Figure** **6**A,B). In HEK293T cells, 4‐HNE directly adducts OTUD5 and GPX4, which induces the carbonylation of GPX4 and OTUD5 (Figure 6C,D; Figure S8A,B, Supporting Information). As ALDH2 is the main metabolic enzyme of 4‐HNE, we subsequently detected that the modification of GPX4 and OTUD5 by 4‐HNE was decreased in HEK293T cells transfected with the ALDH2 plasmid (Figure S8C,D, Supporting Information). This finding prompted us to determine whether GPX4 and OTUD5 were potential targets of 4‐HNE covalent binding in the MI/R model. In ALDH2 cKO mice, co‐immunoprecipitation assays showed that the levels of 4‐HNE adducted to GPX4 and OTUD5 were increased and the binding between GPX4 and OTUD5 was reduced (Figure S8E,F, Supporting Information). Collectively, these data indicated that MI/R enhanced 4‐HNE production and induced GPX4 and OTUD5 carbonylation, especially in ALDH2 deficient mice.

To determine the potential carbonylation sites of GPX4 and OTUD5, we performed liquid chromatography‐mass spectrometry (LC‐MS) analysis and identified cysteine residues (C93 of GPX4 and C247 of OTUD5) modified by 4‐HNE (Figure 6E,F). Next, we constructed carbonylation‐defective GPX4 and OTUD5 mutants with cysteine residue substituted with alanine (C93A and C247A) to further investigate the function of the carbonylation sites. In mutant‐transfected HEK293T cells, the carbonylation of GPX4 and OTUD5 were significantly abolished (Figure 6G), the ability of 4‐HNE to weaken the interaction of GPX4 and OTUD5 and promote GPX4 ubiquitin–proteasomal degradation was eliminated (Figure 6H). N‐acetylcysteine (NAC) contains reactive cysteine, which can inactivate cysteine‐reactive metabolites.^[^ ^30^ ^]^ When 4‐HNE was co‐incubated with NAC in NRCMs, the similar results of carbonylation‐defective were obtained, which suggests that cysteine is the essential residue for 4‐HNE carbonylation (Figure S8G–J, Supporting Information).

### 2.7 Overexpression of OTUD5 Ameliorates MI/R‐Induced Ferroptosis and Myocardial Remodeling

Encouraged by the effect of OTUD5 against GPX4 degradation in vitro, we next engineered adeno‐associated virus 9 (AAV9) viral vectors with a cardiomyocyte‐specific cTnT promoter to overexpress OTUD5 in ALDH2 cKO mice. AAV9‐mediated cardiac‐specific OTUD5 overexpression was confirmed by western blotting in cardiac tissues (Figure S9A, Supporting Information).

**Figure 7:**
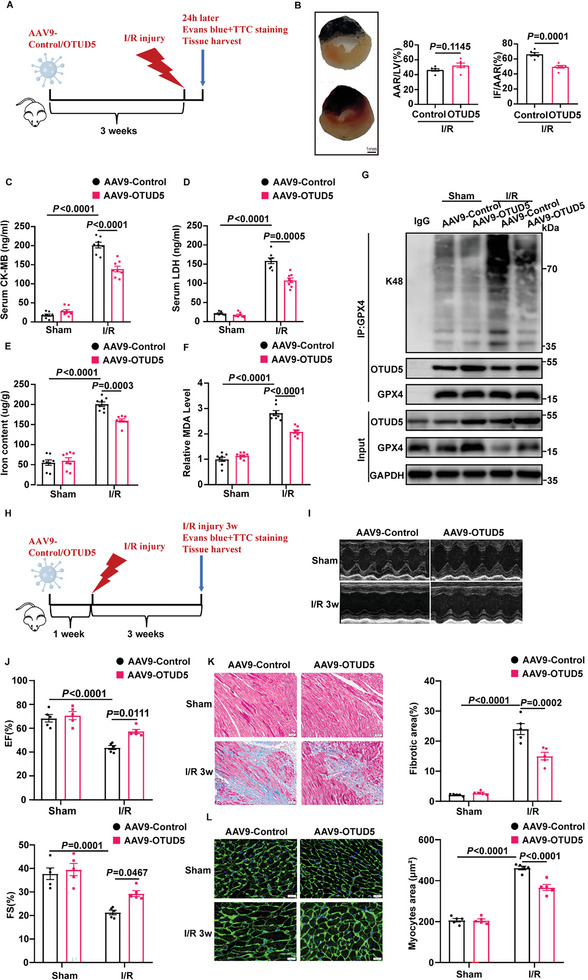
OTUD5 overexpression prevents cardiac ischemia/reperfusion injury in ALDH2 cKO mice. A) Schematic diagram showing that AAV9‐Control or AAV9‐OTUD5 were injected via tail vein, and 3 weeks later mice were subjected to cardiac ischemia/reperfusion (I/R) injury for 24 h. B) Representative sections and quantitative data for infarct size (IF) and area at risk (AAR) in mice hearts subjected to I/R injury (30‐min ischemia/24‐h reperfusion, n = 6). Scale bar: 1 mm. C,D) Serum levels of CK‐MB and LDH in mice with sham or MI/R injury (30‐min ischemia/24‐h reperfusion, n = 8). E,F) Iron content and MDA levels in mice subjected to MI/R (30‐min ischemia/24‐h reperfusion, n = 8). G) The ubiquitination of GPX4 was determined by immunoprecipitation with anti‐GPX4 antibody in mice subjected to MI/R injury (30‐min ischemia/24‐h reperfusion, n = 4). H) Schematic diagram showing that AAV9‐Control or AAV9‐OTUD5 were injected via tail vein, and 1 week later mice were subjected to cardiac ischemia/reperfusion (I/R) injury for 3 weeks. I,J) Echocardiography for left ventricular ejection fraction (EF, %) and fractional shortening (FS, %) in mice after I/R injury (30‐min ischemia/3‐week reperfusion, n = 5). K,L) Masson Trichrome staining for cardiac fibrosis (Scale bar: 50 µm) and WGA staining for cardiac hypertrophy (Scale bar: 20 µm) in heart tissues of mice after MI/R (30‐min ischemia/3‐week reperfusion, n = 5). Data are expressed as mean ± SEM. Unpaired two‐tailed Student's t‐test was used for the analysis in B). One‐way ANOVA was used for the analysis in C–F,J–L).

To study the functional roles of OTUD5 in vivo, mice injected with AAV9 expressing OTUD5 (AAV9‐OTUD5) were subjected to acute cardiac I/R injury (**Figure** **7**A). As predicted, AAV9‐OTUD5 injected mice exhibited a decreased response to I/R‐induced myocardial injury, as revealed by the decrease in infarct size and CK‐MB and LDH levels (Figure 7B–D). To evaluate whether OTUD5 inhibited ferroptosis, we assessed the levels of iron, MDA, and GSH (Figure 7E,F; Figure S9B, Supporting Information). The results showed that OTUD5 overexpression restored the increase in iron and MDA levels and the decrease in GSH caused by MI/R. Moreover, ubiquitination of GPX4 was evidently increased in I/R‐treated mouse hearts, which was recovered by AAV9‐OTUD5 injection (Figure 7G). These data implied that the protective effect of OTUD5 against I/R‐induced myocardial injury may be related to GPX4 dysfunction‐mediated ferroptosis. To further investigate whether OTUD5 overexpression could protect the heart in the long term, we injected mice with AAV9‐OTUD5 and then subjected them to cardiac I/R injury for 3 weeks (Figure 7H). At 3 weeks post I/R injury, mice injected with AAV9‐OTUD5 had well‐preserved cardiac function compared with the AAV9‐Control‐injected I/R group (Figure 7I,J; Table S3, Supporting Information). Meanwhile, OTUD5 overexpression reduced cardiac fibrotic areas (Figure 7K) and cardiac hypertrophy (Figure 7L). These data indicate that OTUD5 overexpression is adequate to prevent I/R‐induced cardiac dysfunction and maladaptive remodeling.

The aforementioned data indicate that OTUD5 may function as a protective factor in myocardial injury induced by both acute and chronic cardiac ischemic insults. Taken together, we conclude that OTUD5 overexpression offers a safer and more effective treatment for I/R‐induced heart disease.

### 2.8 4‐HNE Accumulation and GPX4 Reduction were Corroborated in Human Ischemic Samples

To confirm the above findings in the clinical situation, we collected heart samples from patients who underwent coronary artery bypass graft surgery and compared them with those from mitral valve replacement surgery. The demographic and clinical data of the patients are presented in Table S4 (Supporting Information).

**Figure 8:**
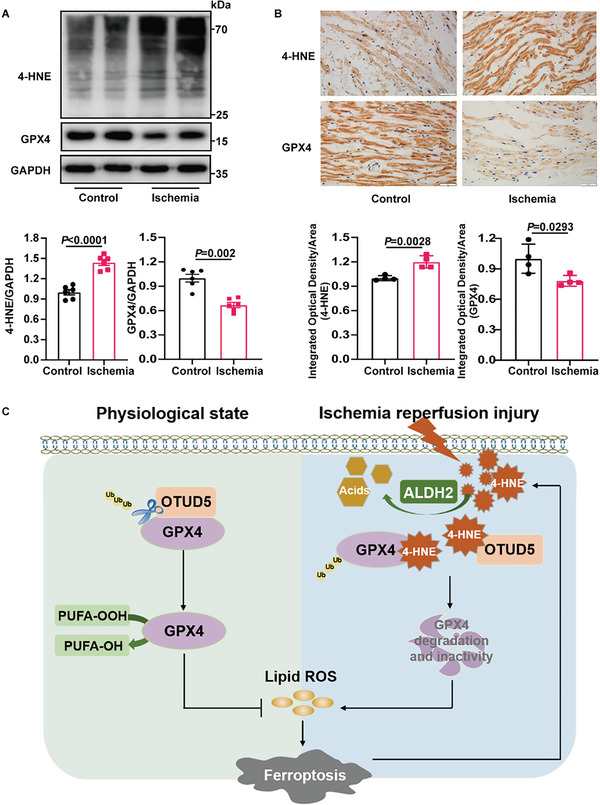
Myocardial ischemia induces 4‐HNE accumulation and GPX4 reduction in human samples. A) Immunoblots of 4‐HNE and GPX4 proteins in the right auricle from control patients and patients with ischemia (n = 6). B) Representative immunohistochemical staining of the control and ischemic patients (n = 4). Scale bar:50 µm. C) Schematic illustration of 4‐HNE‐induced ferroptosis in myocardial ischemia‐reperfusion. At basal conditions, OTUD5 deubiquitinates and stabilizes GPX4. In myocardial ischemia/reperfusion, 4‐HNE binds competitively to OTUD5 and GPX4, blocks the interaction between OTUD5 and GPX4, and promotes GPX4 ubiquitination and degradation, which induces ferroptosis and aggravates myocardial injury. Data are expressed as mean ± SEM. Unpaired two‐tailed Student's t‐test was used for the analysis in A,B).

Interestingly, the results were highly consistent with the above findings obtained in mouse hearts. As shown in **Figure** **8**A,B, 4‐HNE expression was markedly increased and GPX4 levels were significantly decreased in atrial tissues from coronary artery bypass graft patients. Collectively, these data demonstrate the clinical relevance of 4‐HNE and GPX4 in patients with ischemic heart disease and suggest that they may serve as novel therapeutic targets.

## 3 Discussion

Ferroptosis occurs during MI/R injury and is accompanied by the accumulation of 4‐HNE. Here, we provide multiple lines of evidence that 4‐HNE plays an initiating role in MI/R‐induced myocardial ferroptosis. Cardiac‐specific deletion of ALDH2, which dissipates 4‐HNE, aggravates myocyte ferroptosis, whereas ALDH2 activation attenuates ferroptosis. Mechanistically, 4‐HNE triggers GPX4 K48‐linked polyubiquitination and subsequent proteasomal degradation, via directly binding to GPX4 and OTUD5 at cysteine residues. 4‐HNE is not only a byproduct of ferroptosis but also constitutes a positive feedback loop for exacerbating myocyte ferroptosis via the 4‐HNE‐GPX4 axis. Moreover, the accumulation of 4‐HNE and reduced expression of GPX4 were confirmed in human ischemic heart samples. Altogether, we found that accumulation of 4‐HNE during myocardial I/R leads to ferroptosis through disrupting GPX4. 4‐HNE can also cause an increase in iron content, and the underlying mechanism requires to be studied further.

Preventing cardiac cell death is an effective cardioprotective strategy in myocardial diseases.^[^ ^31^ ^]^ Multiple studies have identified 4‐HNE, which is abundantly produced during MI/R, as a major contributor to cell death. Our previous study showed that the clearance of 4‐HNE protected against MI/R injury by restraining necroptosis.^[^ ^20^ ^]^ In this study, we further verified that 4‐HNE provoked myocyte ferroptosis in MI/R insults. Cardiac‐specific knockout of ALDH2, the key metabolic enzyme of 4‐HNE, aggravated 4‐HNE accumulation and ferroptosis, whereas the activation of ALDH2 exerted opposite effects. In vitro, 4‐HNE stimulation triggers cardiomyocyte ferroptosis, and detoxifying 4‐HNE or a ferroptosis inhibitor blocks the deleterious effects of 4‐HNE. Our findings are consistent with those of Chen et al.,^[^ ^32^ ^]^ who showed that supraphysiological levels of 4‐HNE could trigger ferroptosis in HT‐1080 and Calu‐1 tumor cells, although the underlying molecular mechanisms have not been elucidated. These findings indicate that 4‐HNE is not only a byproduct of ferroptosis but also an inducer of ferroptosis. In addition, given that cellular necroptosis and ferroptosis have been reported to interact with each other to synergistically exacerbate cell death and accelerate the progression of diseases,^[^ ^33^, ^34^, ^35^ ^]^ 4‐HNE may be an important regulator connecting these two forms of cell death. Targeting the reduction in 4‐HNE is expected to be a promising treatment for MI/R injury. However, specific effective clinical interventions for reducing 4‐HNE levels are still lacking. Hence, exploring the crucial molecules involved in 4‐HNE‐triggered ferroptosis may provide a new approach to the prevention of MI/R injury.

To date, three major pathways have been implicated in ferroptosis: canonical glutathione‐GPX4 ferroptosis‐controlling axis, the ferroptosis suppressor protein1(FSP1)–ubiquinone system, and squalene‐mediated and di/tetrahydrobiopterin (BH2/BH4)‐mediated inhibition of lipid peroxidation.^[^ ^36^ ^]^ The proper functioning of GPX4 is a central suppressor of ferroptosis, whereas inhibition or destabilization of GPX4 sensitizes or even triggers ferroptotic cell death. It has been reported that DMOCPTL (a derivative of the natural product parthenolide) and Bufotalin (a natural small molecule) can facilitate the degradation of GPX4 and trigger ferroptosis in cancer cells.^[^ ^37^, ^38^ ^]^ In addition, FINO2 (an endoperoxide‐containing 1,2‐dioxolane) and Honokiol (a biphenolic compound) can also initiate ferroptosis by inhibiting the activity of GPX4.^[^ ^39^, ^40^ ^]^ Interestingly, through screening key ferroptosis‐associated molecules, we found that only GPX4 was regulated by 4‐HNE. 4‐HNE contributes to GPX4 dysfunction by promoting GPX4 K48‐linked ubiquitination and degradation. Therefore, it is conceivable that the inhibition of 4‐HNE‐induced GPX4 ubiquitination may alleviate myocardial ferroptosis.

Ubiquitination is a vital post‐translational modification that plays a critical role in the regulation of protein stability. Ubiquitination is primarily mediated by E3 ubiquitin ligase, which acts synergistically with E1 and E2 to promote proteasome‐mediated protein degradation. Previous studies have reported that ubiquitination modification of GPX4 is associated with E3 ubiquitin ligases MIB2 and TRIM26.^[^ ^41^, ^42^ ^]^ Dong et al. found that linear ubiquitin chain assembly complex (LUBAC) performed linear ubiquitination of GPX4 to enhance its stability.^[^ ^43^ ^]^ Notably, ubiquitination is a reversible process under the control of deubiquitinating enzymes, negating the action of E3 and enhancing protein stability. Recently, Li et al. reported that deubiquitinating enzyme OTUB1 inhibited GPX4 ubiquitination and enhanced its protein stability in human gastric cancer cells.^[^ ^44^ ^]^ In our study, we first identified OTUD5 as GPX4 deubiquitinase in cardiomyocytes. Overexpression of OTUD5 blocked the detrimental effects of 4‐HNE on GPX4 ubiquitination and degradation in vivo and in vitro. OTUD5, a member of the ovarian tumor family, has recently emerged as a critical regulator in various diseases and pathological processes, including Primary Biliary Cholangitis,^[^ ^45^ ^]^ embryogenesis,^[^ ^46^ ^]^ inflammatory bowel disease,^[^ ^47^ ^]^ DNA repair,^[^ ^22^ ^]^ and immune response.^[^ ^24^ ^]^ Interestingly, our RNA‐seq data indicated that the deubiquitinase enzyme OTUD5 depletion resulted in increased ferroptosis, and SLC7A11/SLC3A2 complex was significantly decreased in siOTUD5 cardiomyocytes. Ferroptosis regulated by OTUD5 may be not only related to the direct protein ubiquitination degradation of GPX4, but also related to indirect reduction of SLC7A11/SLC3A2 mRNA levels by transcription factors or other transcriptional regulation, which could be an interesting study point in the future. In addition, we have demonstrated that OTUD5 overexpression is able to profoundly mitigate I/R‐induced ferroptosis, together with subsequent myocardial remodeling, suggesting that OTUD5 is a promising new therapeutic option for myocardial infarction patients receiving reperfusion therapy.

4‐HNE, as a diffusible and highly reactive agent, can undergo a Michael addition reaction with nucleophilic residues, including cysteine, histidine, and lysine to form carbonylation modifications, which in turn lead to alterations in protein structure and signaling pathways.^[^ ^48^ ^]^ It can bind to VDAC1 and MCU to promote mitoCa^2+^ overload and mitochondrial dysfunction in chronic postischemic cardiac remodeling.^[^ ^16^ ^]^ Chronic pain‐induced 4‐HNE overload provoked cardiac SIRT1 carbonylative inactivation and inhibited Liver kinase B1 (LKB1)‐AMPK interaction resulting in exacerbated MI/R injury.^[^ ^15^ ^]^ In this study, we found that 4‐HNE directly modified the carbonyl groups of cysteine residues of GPX4 and OTUD5, leading to their impaired activity and interaction. In addition, C93 and C247 are essential residues for 4‐HNE‐induced carbonylation. N‐acetyl cysteine (NAC), an analog of cysteine that competitively binds to 4‐HNE, and amino acid substitution rescued 4‐HNE‐induced cysteine carbonylation of GPX4 and OTUD5, recovered the interaction between GPX4 and OTUD5, alleviated ubiquitination‐dependent degradation of GPX4, and inhibited myocyte ferroptosis. Hence, these data further corroborated that 4‐HNE could initiate ferroptosis directly by its carbonyl‐modifying effects, which further verifies the unique pathogenic role of toxic aldehydes derived from aldehyde metabolism disorder in the development of cardiovascular diseases.

In addition, nearly 8% of the world population and 40% of the East Asian population carry the ALDH2*2 mutant allele,^[^ ^49^, ^50^ ^]^ leading to a reduction in the enzymatic activity for metabolizing 4‐HNE. Our previous studies confirmed that the ALDH2*2 mutant allele is an independent risk factor for the severity of cardiac damage in myocardial infarction patients.^[^ ^51^, ^52^, ^53^ ^]^ It has been reported that ALDH2 overexpression rescues cardiac contractile dysfunction in Alzheimer's disease via inhibiting ferroptosis.^[^ ^54^ ^]^ In the present study, we found that ALDH2 cKO significantly exacerbated myocyte ferroptosis after MI/R by increasing GPX4 and OTUD5 carbonylation, which may be a new molecular mechanism underlying MI/R injury in patients with ALDH2 mutations.

In summary, for the first time, we uncovered the mechanisms by which 4‐HNE initiates myocyte ferroptosis and aggravates MI/R injury. We propose a model whereby MI/R promotes 4‐HNE accumulation, which directly carbonyl‐modifies GPX4 and OTUD5, impairs the interaction of GPX4 with OTUD5, increases GPX4 ubiquitination and degradation, and ultimately triggers cardiomyocyte ferroptosis (Figure 8C). Specific targeting of OTUD5 may mitigate I/R‐induced ferroptosis and cardiac injury. These findings suggest that the 4‐HNE‐GPX4 axis is a key contributor to myocyte ferroptosis through a positive‐feedback loop, which not only reveals the novel molecular mechanisms underlying 4‐HNE‐induced cell death but also identifies OTUD5 as a promising therapeutic target for the treatment of MI/R injury.

## 4 Experimental Section

### Animals and Treatment

The present study was approved by the Animal Ethics Committee of the Qilu Hospital of Shandong University (KYLL‐2022(ZM)−038). ALDH2 cKO mice and their littermates were generated by Cyagen Biosciences. WT mice, ALDH2 cKO mice, and their littermates were used to perform the MI/R injury surgery. After anesthesia with sevoflurane inhalation, the hearts were subjected to thoracotomy. The left anterior descending (LAD) artery was tied with an 8‐0 silk suture for 30 min and loosened for 24 h. Then, the hearts and blood of mice were harvested for the following experiments. The ALDH2 activator Alda‐1 (25 mg kg^−1^) or ferroptosis inhibitor DXZ (50 mg kg^−1^) was administered intraperitoneally.

### Generation of Cardiomyocyte‐Specific ALDH2‐Knockout Mice

ALDH2^flox/flox^ mice, which possess loxP sites flanking exon 2–4 of ALDH2, were generated by Cyagen Biosciences. ALDH2^flox/flox^ mice were crossed with α‐MHC‐Cre mice to generate α‐MHC‐Cre; ALDH2^flox/+^ mice. Finally, α‐MHC‐Cre; ALDH2^flox/+^ mice were crossed to obtain α‐MHC‐Cre; ALDH2^flox/flox^ mice (cardiomyocyte‐specific ALDH2‐knockout mice, ALDH2 cKO) at the predicted Mendelian ratio.

### Cell Culture

Primary neonatal rat cardiomyocytes (NRCMs) were isolated from the hearts of 2‐ to 3‐day‐old Sprague‐Dawley rats as previously described. Cultured NRCMs and H9c2 cells were incubated with Alda‐1 (20uM)/DXZ (10uM) during 6 h of 4‐HNE (40uM) treatment. Cells were transfected with plasmids or siRNAs using Lipofectamine 2000 or RNAiMAX (Invitrogen, USA), according to the manufacturer's instructions.

### Generation of GPX4 and OTUD5 KO Cell Lines

The single guide RNA (sgRNA) sequence targeting GPX4 or OTUD5 and CRISPR/Cas9 lentivirus were produced and used according to the manufacturer's instructions (GeneChem, Shanghai, China). Briefly, 5 × 10^4^ ml^−1^ H9c2 cells were seeded into 6‐well plates the day before transfection. The lentivirus was added to H9c2 cells with 40 µL HitransG A (GeneChem) and 1 mL complete DMEM medium. After 16 h, the medium was replaced with fresh culture medium. Then the stable cell lines were selected using puromycin incubation. GPX4 and OTUD5 knockout was confirmed by western blotting. The sgRNA target sequences were listed in Table S5 (Supporting Information).

### Plasmids, siRNAs, and Transfection

Full‐length cDNA of GPX4, OTUD5, Ub and their mutants, tagged with HA and FLAG, were amplified by standard PCR and subcloned into the pcDNA 3.1 vector, and pcDNA 3.1 plasmids were used as controls. All constructed plasmids were transfected into cells using Lipofectamine 2000 (Invitrogen, USA) following the manufacturer's instructions.

The siRNA targeting GPX4 (siGPX4) and OTUD5 (siOTUD5) were all synthesized by GenePharma (Shanghai, China). The sequences were presented in Table S5 (Supporting Information). Cardiomyocytes were transfected with siRNAs using Lipofectamine RNAiMAX (Invitrogen, USA) according to the manufacturer's protocol.

### Echocardiography

Transthoracic echocardiography was performed using a Vevo3100 system (Fujifilm Visual Sonics, Canada) with a 30‐MHz transducer (MS400). The mice were placed on a heating stable pad, anesthetized with 1–2% isoflurane, and maintained at a heat rate of > 400 beats min^−1^. Echocardiography parameters were obtained from the LV parasternal long and short axes. Three images from consecutive cardiac cycles were analyzed and averaged.

### Infarct Size Measurement

To determine myocardial infarct size, 1% Evans blue dye (EBD) was perfused into the heart through the aorta after the LAD artery was tied at previous place. After that, the hearts were frozen and cut into 1–2 mm slices. Then, the slices were incubated in 1% 2,3,5‐triphenyl tetrazolium chloride (TTC) solution for 15–20 min at 37 °C. After photographing, the images were analyzed using ImageJ software (NIH). The infarct area (pale area) and AAR (pale area plus pink area) were determined by planimetry using the ImageJ software. Infarct size was calculated as the infarct area divided by the AAR.

### CK‐MB and LDH Assay

The CK‐MB and LDH levels were determined using a commercial ELISA kit (Cloud‐Clone Corp, China). Serum was collected at the end of reperfusion and added to the master reaction mix, according to the manufacturer's instructions. The absorbance at 450 nm was then analyzed using a reader.

### Measurement of Iron Content, Malondialdehyde (MDA), Glutathione (GSH), and GPX4 Activity

An Iron Assay Kit (ab83366, Abcam), malondialdehyde (MDA) Assay Kit (Jiancheng, China), total glutathione/oxidized glutathione assay kit (Jiancheng, China), and Glutathione Peroxidase Assay Kit (ab102530, Abcam) were used to determine the relative levels of iron content, MDA, GSH, and GPX4 activity, respectively. All procedures were performed in accordance with the instructions.

### Lipid ROS Assay using Flow Cytometer

Myocardial cells were incubated in DMEM containing 5 µM BODIPY 581/591 C11 for 30 min in a cell culture incubator. The cells were then collected by trypsinization and resuspended in 500 µL of PBS. Lipid ROS levels were analyzed using a Beckman CytoFLEX machine through the FL1 channel. A total of 10000 cells were analyzed for each sample.

### Cell Viability Analysis

Cardiomyocyte viability was assayed using a cell counting kit‐8 (CCK8) in a culture medium. CCK8 was spectrophotometrically measured using a kit (Solarbio, China), according to the manufacturer's instructions.

### Western Blotting and Co‐Immunoprecipitation

The proteins of the harvested heart tissues or cultured cells were sonicated in lysis buffer and quantified using the BCA assay. Protein samples were separated using SDS‐PAGE and transferred to polyvinylidene fluoride (PVDF) membranes (Merck Millipore, Billerica, MA, and USA). The membranes were incubated with primary antibodies overnight at 4° C. After washing, the membranes were incubated with horseradish peroxidase (HRP)‐coupled secondary antibody. Blots were visualized using chemiluminescence reagents and analyzed using the ImageJ software.

Ubiquitination of GPX4 and OTUD5 was assessed by immunoprecipitation. H9c2, NRCMs, and 293T cells were lysed in Cell Lysis Buffer. Then, the protein was incubated with GPX4 and OTUD5 antibodies or IgG overnight, which was followed by 3 h of incubation at 4 °C with 20 µL Protein A/G Mix Magnetic Bead (Merck, United States). Next, 20 µL of loading buffer was added to the beads and boiled. The supernatants were subjected to SDS‐PAGE and analyzed.

### Immunohistochemistry and Immunofluorescence

For immunohistochemistry, the sections were incubated with anti‐4‐HNE and anti‐GPX4 antibodies (Abcam, Cambridge, UK) overnight at 4 °C followed by being washed, and stained with secondary antibodies. Then, 3,3′‐diaminobenzidine (DAB) was used as the chromogenic substrate. The slices were counterstained with hematoxylin.

For immunofluorescence staining, cells grown on a cover glass were immobilized with 4% paraformaldehyde and permeabilized with 0.1% Triton X‐100. After blocking with goat serum, the cells were incubated overnight with primary antibodies. After the cells were washed, the secondary antibody was incubated at 37 °C for 30 min in dark, followed by DAPI incubation to visualize the nuclei.

### Duolink Proximity Ligation In Situ Assay

NRCMs were fixed with 4% paraformaldehyde and permeabilized with 0.1% Triton X‐100. Subsequent blocking, antibody hybridizations, proximity ligations, and detections were performed according to recommendations from manufacturers (Sigma‐Aldrich). The cells were incubated with the primary antibodies overnight at 4 °C. After incubation with primary antibodies, combinations of corresponding in situ proximity ligation assay (PLA) probes for 60 min at 37 °C was applied. The cells were then washed with PBS, incubated for 30 min with ligase, and finally washed with PBS. Then cells were incubated with polymerase for 100 min. Fluorescence was analyzed with a laser confocal microscope.

### GST‐Pull Down Assay

Purified fusion proteins GST‐GPX4 and His‐OTUD5 were expressed in Escherichia coli. For GST‐pull down, the 1 ml GST‐GPX4 fusion protein was mixed with 30 µL of GST beads and incubated at 4 °C overnight. After that, 1 ml of purified OTUD5 protein was added to the mixture of GST‐GPX4 fusion protein and beads with or without 4‐HNE treatment (100 µM for 2 mg ml^−1^ proteome at room temperature for 1 h), and then incubated at 4 °C overnight. The beads were washed 2–3 times with lysis buffer containing PMSF for 20 min each time, and the beads were retained. Then, elution buffer was used to wash the samples. 100 µL of elution buffer was added to each sample, and incubated at 4 °C for 30 min, and the supernatant was collected. Finally, loading buffer was added and boiled, and the samples were analyzed by western blotting.

### Surface Plasmon Resonance (SPR)

The recombinant OTUD5 and GPX4 protein were used for surface plasmon resonance (SPR) analysis using a Biacore T200 instrument (Biacore, Uppsala, and Sweden). The amino group on the protein was coupled to the activated carboxyl group on the CM5 chip. Briefly, 100 µg mL^−1^ of GPX4 in 10 mM NaAc (pH 4.5) was then injected 300s to Fc4 sample channel at a flow rate of 10 µL min^−1^, the immobilization level of about 11 340.4RU. Binding analyses were carried out at 25 °C and a flow rate of 30 µl min^−1^. Dilute OTUD5 with the Running Buffer (1*EP Buffer, GE) to 5 concentrations (50, 25, 12.5, 6.25, 3.125, and 0 nM). OTUD5 is injected to Fc3‐Fc4 of channel at a flow rate of 30 µl min^−1^ for an association phase of 60s, followed by 120 s dissociation. The purpose of OTUD5 Fc3 channel was to rule out the possibility of it binding to the chip surface. The binding curves were analyzed using the steady state affinity model (K*_D_*) supplied with Biacore Evaluation Software (GE Healthcare).

### Microscale Thermophoresis (MST)

Purified His‐OTUD5 protein was labelled using the MO His‐tag Protein Labeling Kit Red‐tris‐NTA (Nanotemper, Germany) following the manufacturer's instructions, and the labelling reaction was performed with 200 nM His‐OTUD5 protein and 100 nM labelling dye. The labelled protein was centrifuged at 15000× g for 10 min at 4 °C before use. Next, the labelled His‐OTUD5 protein was incubated with purified GPX4 protein at different concentrations in PBS‐T buffer. Then, all MST measurements were performed at 25 °C using Monolith NT standard capillaries (NanoTemper Technologies, Germany) and the Monolith device (NanoTemper Technologies) with the laser being on for 5 s using 40% power. All curves were plotted with the MO Affinity Analysis V10 software (NanoTemper Technologies), and the thermophoresis signals were fitted with the Kd model.

### Protein Carbonylation Assay

Protein carbonylation was analyzed using Oxyblotting technology (Millipore, United States). Briefly, the carbonyl groups in the protein side chains were derivatized to 2,4‐dinitrophenylhydrazone by reaction with 2,4‐dinitrophenylhydrazine. The DNP‐derivatized protein samples were immunoprecipitated with the indicated antibodies and analyzed by immunoblotting.

### Validation of 4‐HNE Adduction

Cells overexpressing GPX4 or Flag‐OTUD5 were cleaved to determine the protein concentration. The proteome was normalized to 2 mg ml^−1^, and then treated with 100 µM 4‐HNE for 1 h and labeled with 0.5 mM m‐APA and 60 mM NaBH_3_CN at pH 5.0, for 1 h. Proteomes were precipitated with methanol/chloroform, resuspended in 0.4% SDS/PBS, and followed by a reaction with 100 µM biotin‐BGE2O‐N3, 1 mM TCEP, 100 µM TBTA, and 1 mM CuSO4 for 1 h. After the second precipitation, the samples were immunoprecipitated with anti‐GPX4 and anti‐FLAG antibodies and analyzed by immunoblotting.

### RNA‐Seq Profiling and Analysis

Cardiomyocytes were transfected with siOTUD5 or NC, and the samples were prepared and submitted to LC‐Bio Technology CO., Ltd., (Hangzhou, China) for total RNA isolation, mRNA purification, library preparation, and sequencing. Genes differential expression analysis was performed by DESeq2 software between two different groups (q<0.05 and |Log2FC|> = 1). All differentially expressed genes were used to enrichment analysis of GO functions and KEGG pathways, which were compared to the genome background and defined by hypergeometric test (p<0.05). All genes were performed gene set enrichment analysis using software GSEA (v4.1.0) and MSigDB to identify whether specific GO terms or KEGG pathways show significant difference in two groups (|NES|>1, q<0.05). Among differentially expressed genes, ferroptosis related genes were chose to generate hierarchical clustering heat map with the ggplot library.

### LC‐MS

The proteins in the gel were hydrolyzed using trypsin. In the LC‐MS/MS analysis, the digested products were separated by 120 min gradient elution at a flow rate of 0.300 µL min^−1^ using a Thermo Ultimate 3000 nano‐UPLC system, which was directly interfaced with the Thermo Fusion LUMOS mass spectrometer. The analytical column used was an Acclaim PepMap RSLC column (75 µm ID, 250 mm length, C18). Mobile phase A consisted of 0.1% formic acid and mobile phase B consisted of 100% acetonitrile and 0.1% formic acid. The Fusion LUMOS mass spectrometer was operated in data‐dependent acquisition mode using Xcalibur 4.1.50 software, and there was a single full‐scan mass spectrum in the Orbitrap (375–1500 m z^−1^, 60000 resolution) followed by data‐dependent MS/MS scans. The MS/MS spectra from each LC‐MS/MS run were searched against the selected database using the Proteome Discovery software (version 2.4).

### Human Subjects

Human right atrium samples from patients who underwent coronary artery bypass graft surgery were included in the ischemic group, and age‐matched patients who underwent mitral valve replacement surgery were included as controls. All the participants provided written informed consent. This study was approved by the Medical Institutional Ethics Committee of Qilu Hospital of Shandong University (KYLL‐202011‐199).

### Statistical analysis

All data were shown as the mean ± SEM. Normal distribution was initially analyzed, and then the Brown–Forsythe test was used for the analysis of homogeneity of variance. Comparisons between the two groups were performed using the unpaired two‐tailed Student's t‐test. One‐way analysis of variance (ANOVA) followed by Tukey's post hoc test was used to compare differences between more than two groups. When the data did not pass the normality test, they were analyzed using the Mann‐Whitney test or Kruskal‐Wallis test, followed by the Dunn post hoc test. Statistical analyses were performed using GraphPad Prism 8 (GraphPad, La Jolla, CA, USA). All group numbers and detailed significance values were presented in the figure or their legends.

## Conflict of Interest

The authors declare no conflict of interest.

## Author Contributions

L.L., J.P., and D.Q. contributed equally to this work. L.L., J.P., and D.Q. are the co‐first authors. L.X., F.X. and Y.C. contributed to the study design; L.L. J.P. and D.Q. performed most of the experiments and wrote the paper; R.L., H.Y. and D.Z. provided technical support for animal experiments; W.W., H.R., K.L, and J.W. performed part of molecular biology experiments; K.C. and P.X. participated in protein interaction studies; W.Z., X.S. and X.W collected clinical specimens; Y.C. and J.W. revised the manuscript paper. All authors read and approved the final manuscript.

## Supporting information

Supporting Information

## Data Availability

The data that support the findings of this study are available in the supplementary material of this article.
