# Supplementary material for: Deubiquitinase OTUD5 as a Novel Protector against 4‐HNE‐Triggered Ferroptosis in Myocardial Ischemia/Reperfusion Injury
Source: Adv Sci (Weinh). 2023 Aug 8;10(28):2301852. doi: 10.1002/advs.202301852 (PMC10558642; doi:10.1002/advs.202301852)
Supplement: Supplementary file 1 — Supporting Information [file ADVS-10-2301852-s001.pdf]

ADVANCED SCIENCE
Open Access

Supporting Information

for *Adv. Sci.*, DOI 10.1002/advs.202301852

Deubiquitinase OTUD5 as a Novel Protector against 4-HNE-Triggered Ferroptosis in Myocardial Ischemia/Reperfusion Injury

Lulu Liu, Jiaojiao Pang, Dandan Qin, Ruochuan Li, Dan Zou, Kai Chi, Wenxiao Wu, Haiying Rui, Huaxiang Yu, Wenyong Zhu, Kai Liu, Xuting Wu, Jinxin Wang, Ping Xu, Xiaoshuai Song, Yihai Cao, Jiali Wang, Feng Xu, Li Xue* and Yuguo Chen**

Supporting Information

Deubiquitinase OTUD5 As a Novel Protector Against 4-HNE-Triggered Ferroptosis in Myocardial Ischemia/Reperfusion Injury

Lulu Liu†, Jiaojiao Pang†, Dandan Qin†, Ruochuan Li, Dan Zou, Kai Chi, Wenxiao Wu, Haiying Rui, Huaxiang Yu, Wenyong Zhu, Kai Liu, Xuting Wu, Jinxin Wang, Ping Xu, Xiaoshuai Song, Yihai Cao, Jiali Wang, Feng Xu, Li Xue*, Yuguo Chen**

***Correspondence:** Feng Xu, Li Xue, Yuguo Chen

†**These authors contributed equally:** Lulu Liu, Jiaojiao Pang, Dandan Qin

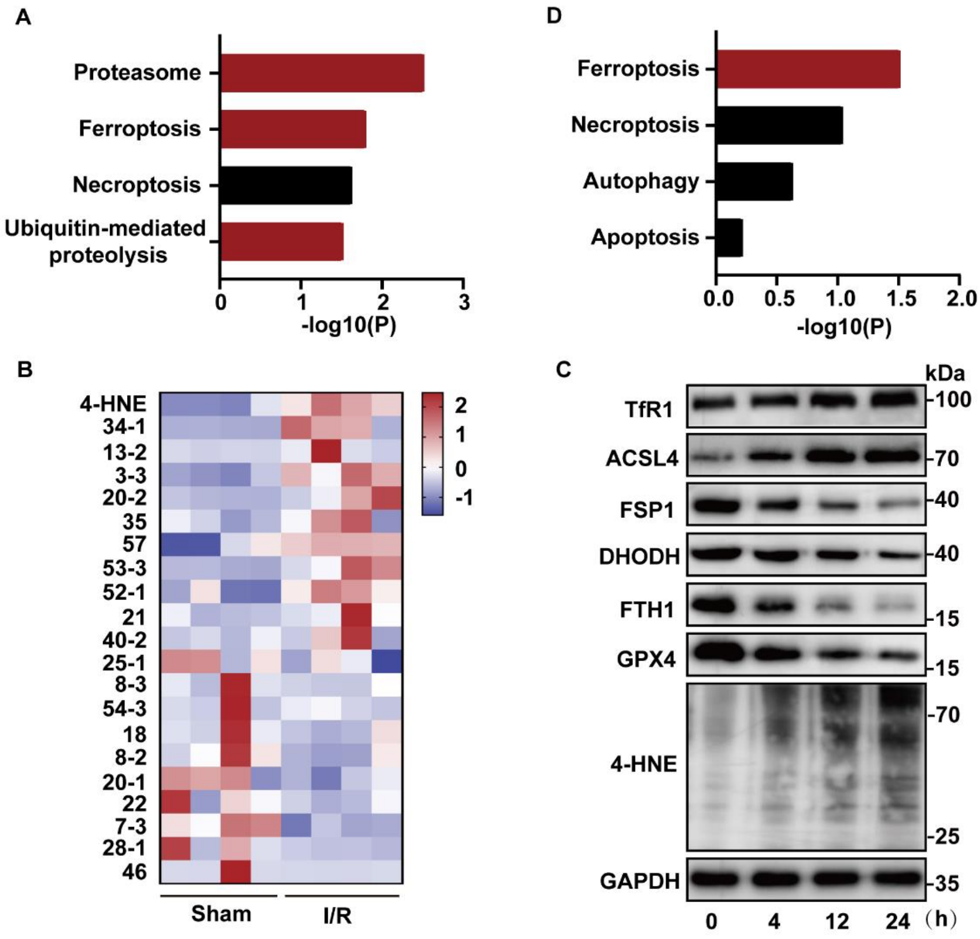

Figure S1. MI/R induces ferroptosis and 4-HNE accumulation.

A, The Kyoto Encyclopedia of Genes and Genomes (KEGG) pathway analysis of RNA-seq data from the hearts of mice subjected to MI/R (n=4).

B, Heatmaps of the fatty aldehydes quantified by high-performance liquid chromatography-multiple reaction monitoring analysis in mice hearts subjected to MI/R injury (n=4).

C, Representative western blotting of 4-HNE and ferroptosis-related proteins in heart tissues. The hearts were collected from mice subjected to I/R surgery after reperfusion at serial time points: 0 hour (sham), 4 hours, 12 hours, 24 hours, (n=4).

D, In the Kyoto Encyclopedia of Genes and Genomes (KEGG) analysis of GSE59100 gene expression data, cell death-related pathways were enriched in ALDH2 mutant ischemic group (n=2)**.**

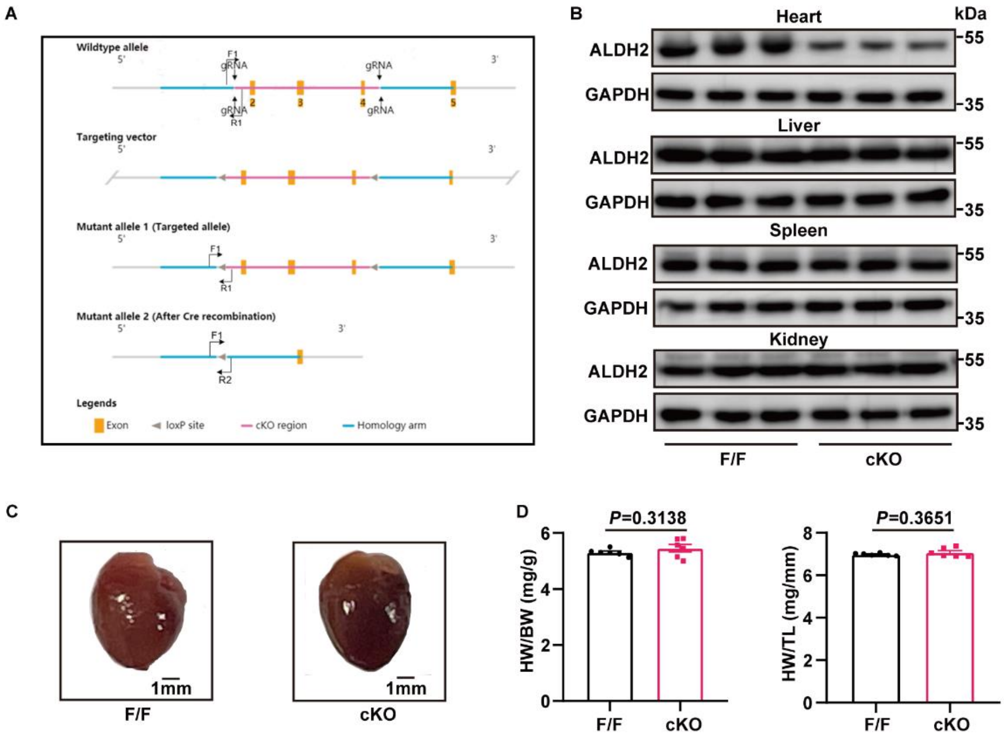

Figure S2. The phenotypes of ALDH2 cKO mice.

A, Schematic diagram showing the breeding strategy of ALDH2 cKO mice. On introduction of cre-recombinase (α-MHC-cre), exon 2-4 of ALDH2 gene was excised specifically in cardiomyocytes of ALDH2$^{flox/flox}$ (F/F) mice, allowing for generation of cardiomyocyte-specific ALDH2-knockout mice (ALDH2 cKO).

B, Representative western blotting of ALDH2 in major organs of F/F and cKO mice (n=4).

C, Gross images of the F/F and cKO mice hearts (n=6). Scale bar:1 mm.

D, Ratio of heart weight/body weight (HW/BW) and heart weight/tibia length (HW/TL) in F/F and cKO mice (n=6).

Data are expressed as mean ± SEM. Unpaired two-tailed Student's t-test was used for the analysis in (D).

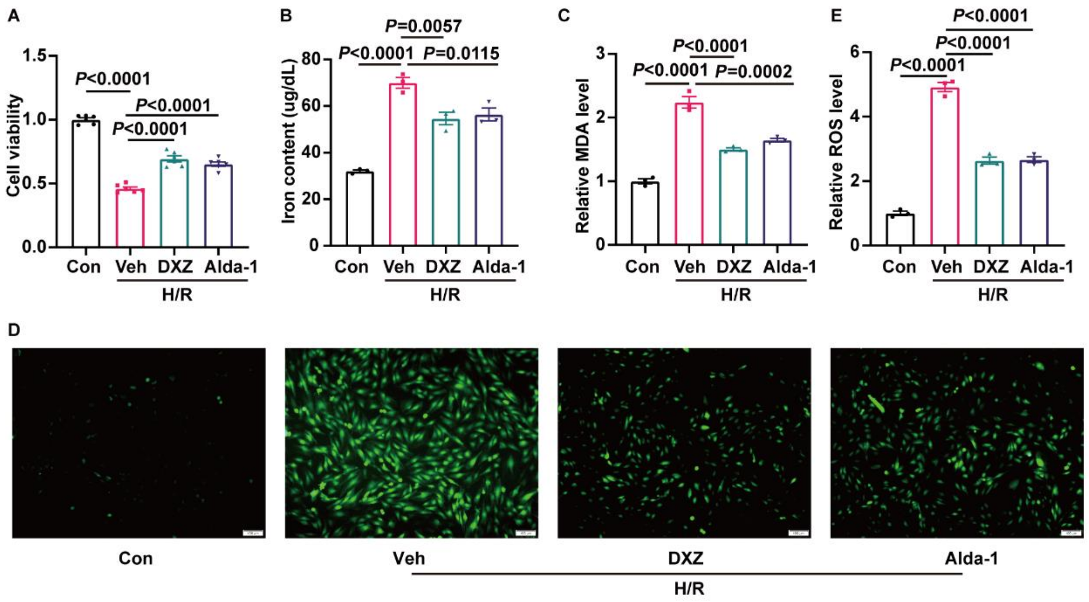

Figure S3. ALDH2 activation rescues ferroptosis in H/R-induced cardiomyocyte injury.

A, Cell viability was measured in H9c2 cells treated with DXZ (10 μM) or Alda-1 (20 μM) during hypoxia/ reoxygenation (n=6).

B-E, The levels of Iron content, MDA, and ROS levels (Scale bar:100 μm) in H9c2 cells treated with DXZ (10 μM) or Alda-1 (20 μM) during hypoxia/ reoxygenation (n=3).

Data are expressed as mean ± SEM. One-way ANOVA was used for the analysis in (A-C, E).

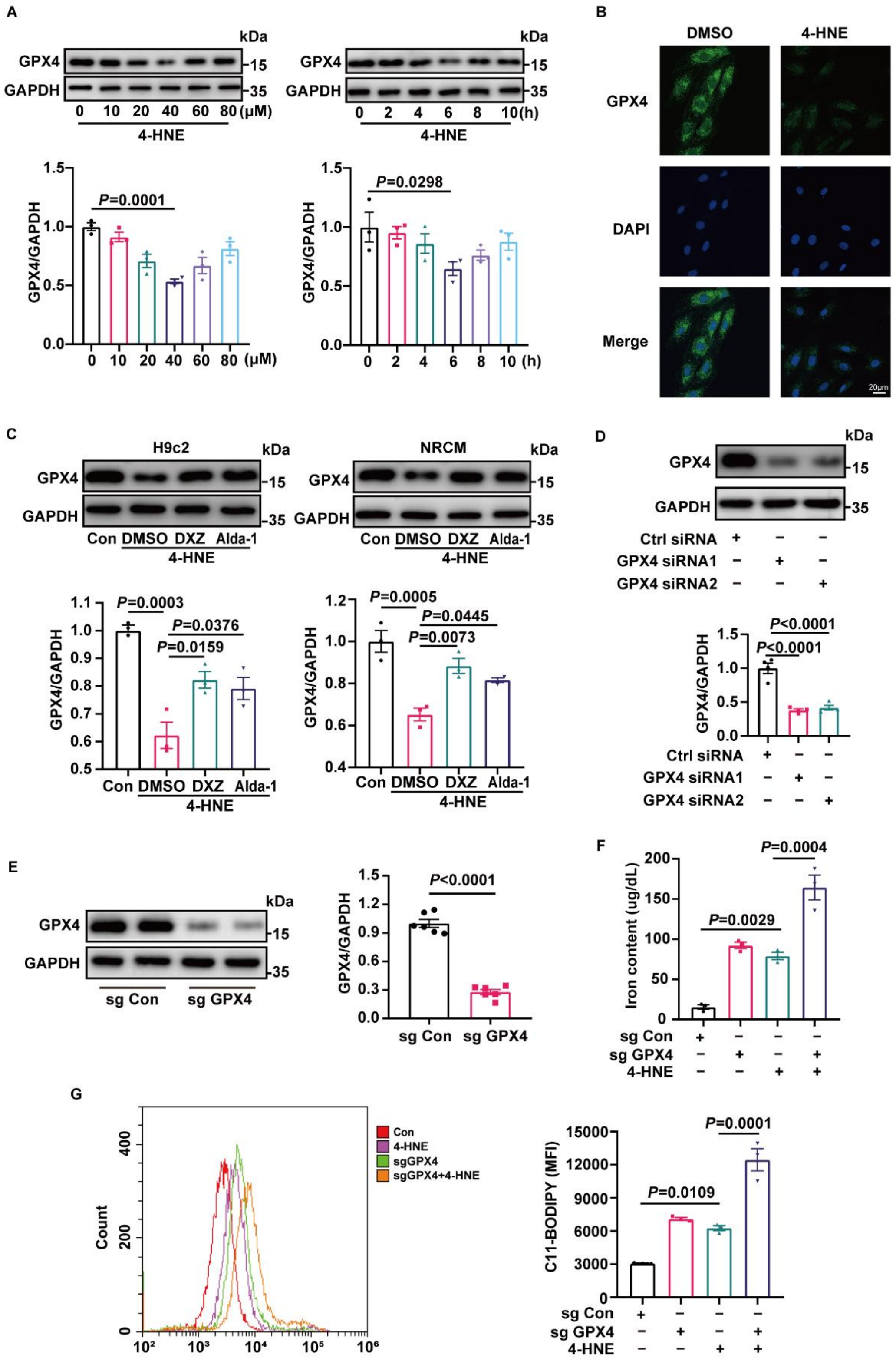

Figure S4. 4-HNE reduces the expression of GPX4.

A, Western blotting analysis of GPX4 proteins in NRCMs under different doses of 4-HNE treatment for 6 h, or under constant 4-HNE treatment (40 μM) for different hours (n=3).

B, Immunofluorescence intensity of GPX4 under 4-HNE treatment (40 μM, 6 h) in H9c2 cells (n=5). Scale bar:20 μm.

C, The expression of GPX4 in H9c2 cells and NRCMs under indicated treatments (n=3).

D-E, Western blotting analysis showing the protein levels of GPX4 transfected with GPX4 siRNAs (n=4) or GPX4 sgRNAs (n=6).

F-G, Iron contents and lipid ROS levels in GPX4 WT (sg Con) and KO (sg GPX4) H9c2 cells after treated with 4-HNE (40 μM,6 h) (n=3).

Data are expressed as mean ± SEM. Unpaired two-tailed Student's t-test was used for the analysis in (E). One-way ANOVA was used for the analysis in (A, C-D, F-G).

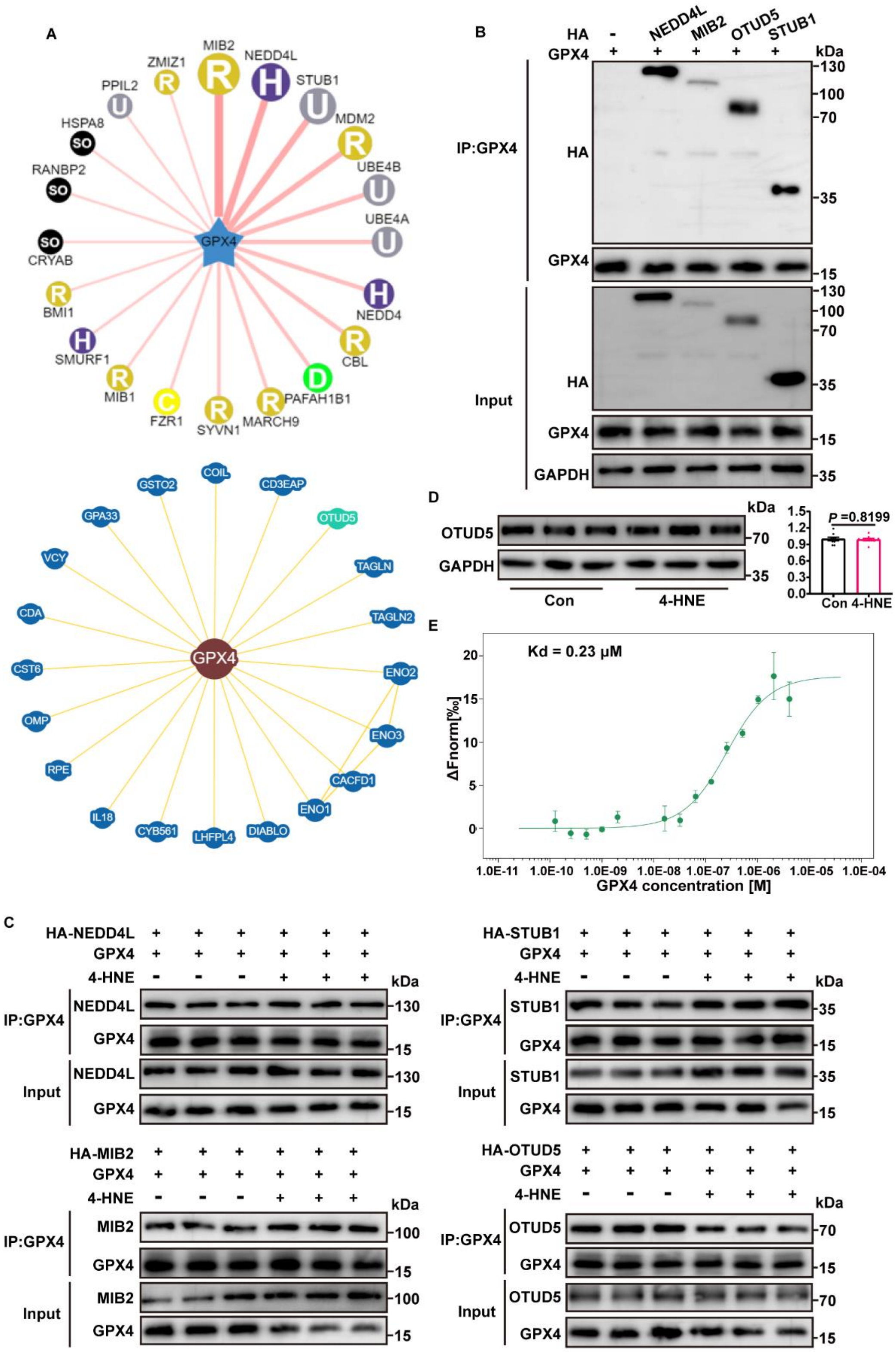

Figure S5. Screen ubiquitination-related enzymes targeting GPX4.

A, Ubibrowser (http://ubibrowser.ncpsb.org/) and Biogrid (https://thebiogrid.org/) were used to predict GPX4 ubiquitin ligase.

B-C, GPX4, HA-NEDD4L, HA-MIB2, HA-OTUD5, and HA-STUB1 were transfected into HEK293T cells with or without 4-HNE incubation (40 μM,6 h),

and Co-IP assays were performed using anti-GPX4 antibody (n=3).
D, The expression of OTUD5 in NRCMs under 4-HNE treatments (40 μM,6 h) (n=9).
E, Purified OTUD5 protein was incubated with increasing concentrations of GPX4 protein, specific interactions were quantified by MST and plotted with the dissociation constant (Kd) equation.
Data are expressed as mean ± SEM. Unpaired two-tailed Student's t-test was used for the analysis in (D).

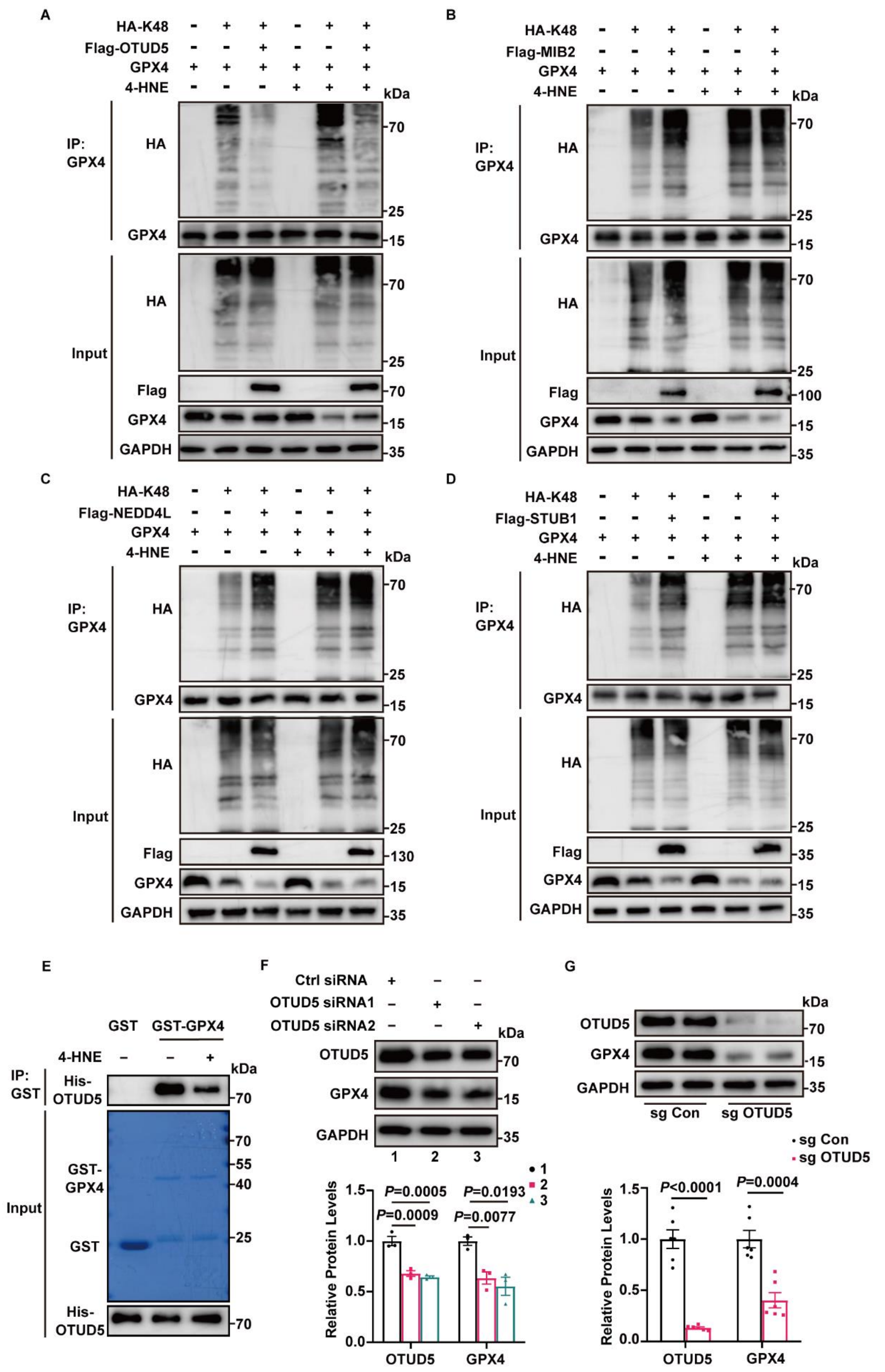

Figure S6. OTUD5 regulates the deubiquitylation of GPX4.

A-D, GPX4 and HA-K48 were transfected into HEK293T cells with or without Flag-OTUD5/MIB2/NEDD4L/STUB1. Cells were treated with 4-HNE (40 μM,6

h), and immunoprecipitation assays were performed (n=3).

E, Recombinant His-OTUD5 was respectively incubated with GST or GST-GPX4 in the presence or absence of 4-HNE before pulldown assay with GST beads (n=3).

F-G, Representative western blotting and averaged data showing the protein levels of GPX4 and OTUD5 in NRCMs transfected with control siRNA (NC) or OTUD5 siRNAs (n=3) or in OTUD5 WT (sg Con) and KO (sg OTUD5) H9c2 cells (n=6).

Data are expressed as mean ± SEM. One-way ANOVA was used for analysis in (F-G).

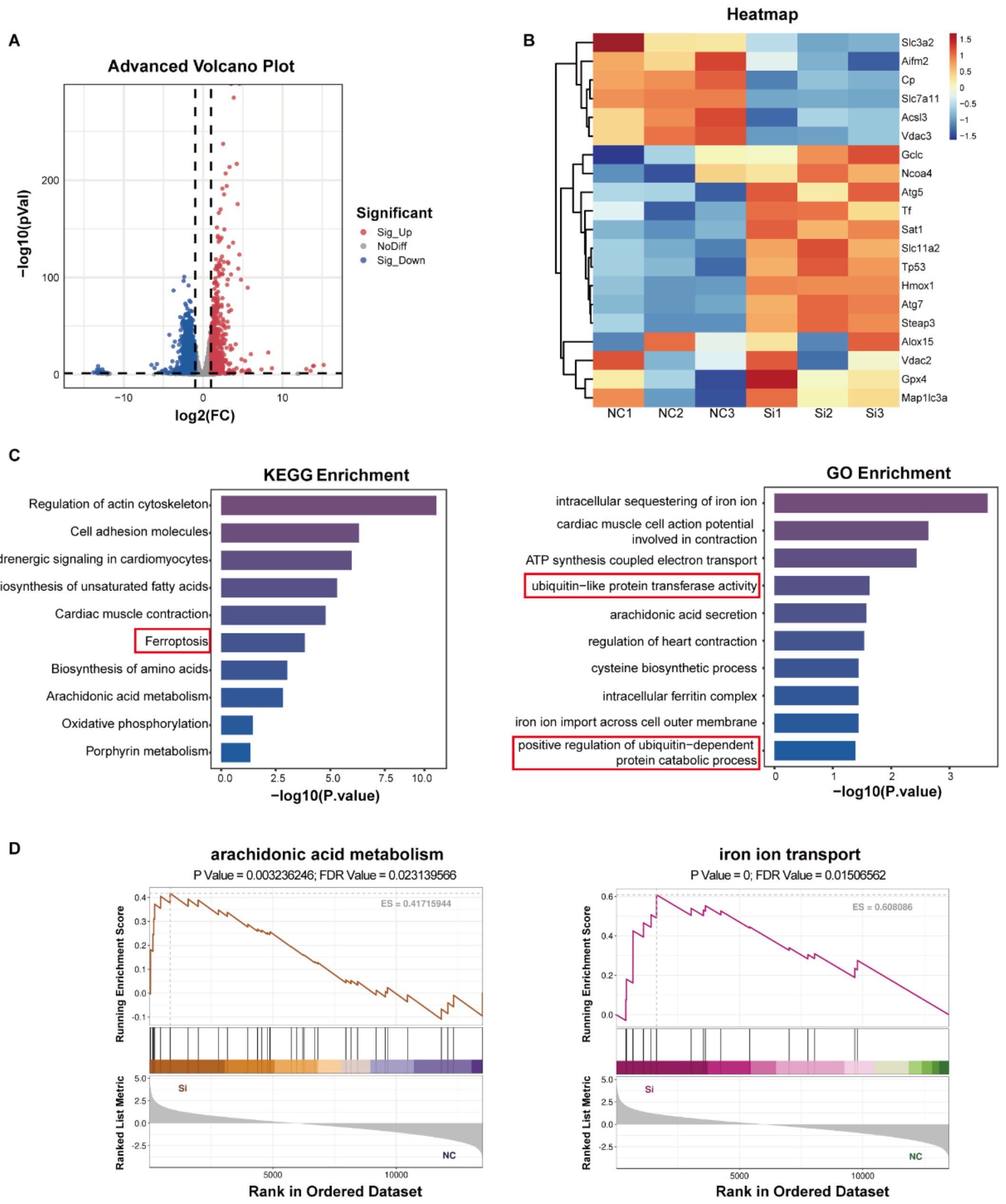

Figure S7. RNA-seq analysis reveals the function of OTUD5 on ferroptosis.

A, Volcano plot for differentially expressed genes. A total of 2904 differentially expressed genes were identified with 863 genes upregulated and 2041 downregulated ($q<0.05$ and $Log_2FC>=1$) (n=3).
B, Heatmap depicting the differentially expressed ferroptosis-related genes of NRCMs in OTUD5 silencing group and control group (n=3).
C, Kyoto Encyclopedia of Genes and Genomes (KEGG) and Gene Ontology (GO) analysis showed that ferroptosis, ubiquitin mediated proteolysis, and cardiomyocyte function were enriched based on the differentially expressed genes of the two groups (n=3).
D, Gene set enrichment analysis (GSEA) of arachidonic acid metabolism and iron ion transport in the expression profiles of NRCMs transfected with OTUD5 siRNAs (n=3).

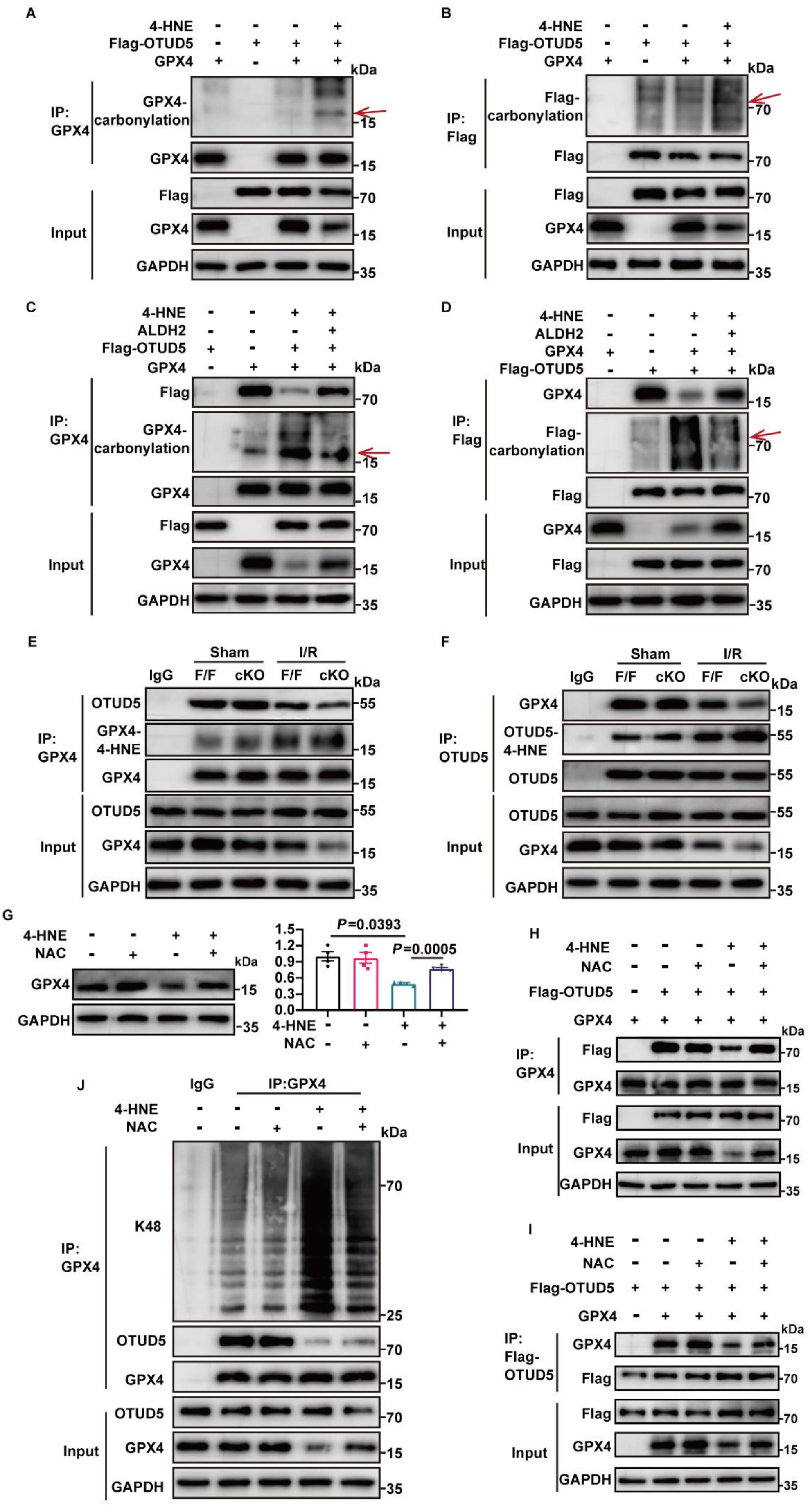
A
4-HNE
Flag-OTUD5
GPX4
kDa
IP: GPX4
GPX4-carbonylation
GPX4
Input
Flag
GAPDH
B
Flag-carbonylation
IP: Flag
C
ALDH2
D
E
Sham
I/R
IgG
F/F
cKO
OTUD5
GPX4-4-HNE
F
OTUD5-4-HNE
IP: OTUD5
G
NAC
P=0.0393
P=0.0005
H
I
IP: Flag-OTUD5
J
IP:GPX4
K48

Figure S8. 4-HNE induces GPX4 and OTUD5 carbonylation.

A-B, Carbonylation assays of GPX4 and OTUD5 in HEK293T cells using oxyblot technology (n=3). The targeted protein bands are indicated by the red arrows.

C-D, Carbonylation assays of GPX4 and OTUD5 by oxyblot technology in HEK293T cells transfected with ALDH2 plasmids (n=3). The targeted protein bands are indicated by the red arrows.

E-F, Representative immunoblots showing the interaction between 4-HNE and GPX4, OTUD5 in F/F and cKO mice after MI/R (30-min ischemia/24-h reperfusion, n=4).

G, Levels of GPX4 protein expression in NRCMs treated with 4-HNE (40 μM) and NAC (0.5 mM) for 6h (n=4).

H-I, Co-immunoprecipitation was performed with GPX4 or Flag antibodies in HEK293T cells treated with 4-HNE (40 μM) and NAC (0.5 mM) for 6h (n=3).

J, Co-IP assays were performed using anti-GPX4 antibody in NRCMs treated with 4-HNE (40 μM) and NAC (0.5 mM) for 6h, and the levels of GPX4 ubiquitination were examined by western blotting (n=3).

Data are expressed as mean ± SEM. One-way ANOVA was used for the analysis in (G).

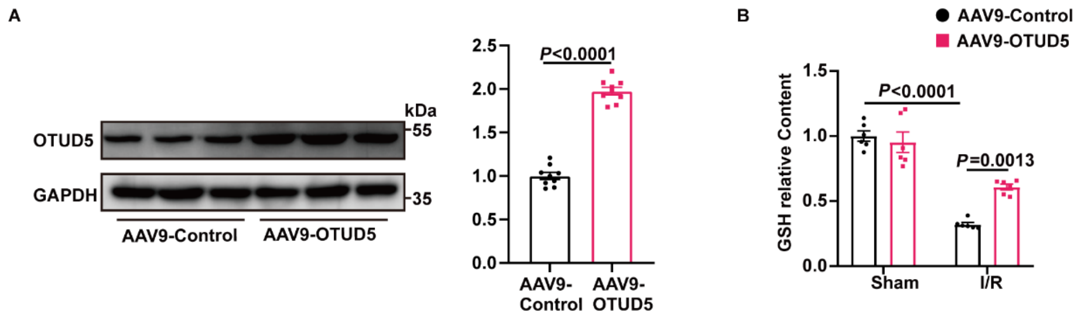

Figure S9. OTUD5 overexpression reversed MI/R-induced the decrease of GSH.

A, Representative immunoblots of OTUD5 in mice hearts treated with AAV9-Control or AAV9-OTUD5 (n=9).

B, GSH levels in AAV9-Control or AAV9-OTUD5 mice subjected to MI/R (30-min ischemia/24-h reperfusion, n=8).

Data are expressed as mean ± SEM. Unpaired two-tailed Student's t-test was used for the analysis in (A). One-way ANOVA was used for the analysis in (B).

Table S1. Echocardiographic analysis of F/F and cKO mice at baseline.

Group	F/F	cKO	*P-value*
Age(week)	8-10	8-10	
n	5	5	
Heart Rate（BMP）	460.40±9.46	462.80±10.03	0.8683
LVESD (mm)	1.99±0.13	2.09±0.16	0.6661
LVEDD (mm)	3.28±0.14	3.39±0.14	0.5999
LVESV(μL)	13.05±2.07	14.76±2.73	0.6316
LVEDV(μL)	43.99±4.44	47.45±4.53	0.6007
EF (%)	71.05±2.01	69.81±3.20	0.7507
FS (%)	39.41±1.59	38.67±2.58	0.8136
LVAW, s (mm）	1.51±0.08	1.58±0.11	0.6105
LVAW, d (mm）	0.99±0.05	1.13±0.12	0.2742
LVPW, s (mm）	1.47±0.08	1.45±0.09	0.8701
LVPW, d (mm）	0.98±0.08	0.97±0.07	0.8934

Data are expressed as mean±SEM.

BPM=beat per minute; LVESD=left ventricular end-systolic diameter; LVEDD =left ventricular end-diastolic diameter; LVESV=left ventricular end-systolic volume; LVEDV=left ventricular end-diastolic volume; EF=ejection fraction; FS=fractional shortening; LVAW, s=left ventricular anterior wall dimension at systole; LVAW, d=left ventricular anterior wall dimension at diastole; LVPW,s= left ventricular posterior wall dimension at systole; LVPW, d=left ventricular posterior wall dimension at diastole.

Table S2. OTUD5 peptides identified in GPX4 complex by mass spectrometry.

Protein name	Confidence	Number of peptides	Number of unique peptides	Coverage [%]	Protein score
OTUD5	High	39	39	53	1869.86

Sequence of unique peptides					
1	QAPGVGAVGGGSPER	14	TSEESWIEQQMLEDK	27	ATSPLVSLYPALECR
2	QAPGVGAVGGGSPEREEVGAGYNSEDEYEAAAAR	15	DRDSGVVGARPR	28	IEAMDPATVEQQEHWFEK
3	NAIKTSEESWIEQQMLEDKK	16	DSGVVGARPR	29	GFIIKQMKEDGACLFR
4	NAIKTSEESWIEQQMLEDK	17	EDGACLFR	30	GFIIKQMK
5	NIHYNSVVNPNK	18	ESYLQWLRDQEKQAR	31	GGGVGVGGGGTGVGGGDRDRDSGVVGARPR
6	RQAPGVGAVGGGSPEREEVGAGYNSEDEYEAAAAR	19	ESYLQWLRDQEK	32	GGGVGVGGGGTGVGGGDRDR
7	RGGGVGVGGGGTGVGGGDRDR	20	ESYLQWLR	33	GGGVGVGGGGTGVGGGDR
8	RGGGVGVGGGGTGVGGGDR	21	ASATCSSATAAASSGLEEWTSR	34	KGFIIK
9	QMKEDGACLFR	22	ATDWEATNEAIEEQVAR	35	KKPPPPDADPANEPPPPGPMPPAPR
10	RATDWEATNEAIEEQVAR	23	ASPPPQGPLPGPPGALHR	36	KHCMDYLMK
11	VSYHRNIHYNSVVNPNK	24	AVADQVYGDQDMHEVVRK	37	KASATCSSATAAASSGLEEWTSR
12	TSEESWIEQQMLEDKKR	25	AVADQVYGDQDMHEVVR	38	KPPPPDADPANEPPPPGPMPPAPR
13	TSEESWIEQQMLEDKK	26	ATIGVGLGLPSFKPGFAEQSLMK	39	HCMDYLMK

Table S3. Echocardiographic analysis of AAV9-Control and AAV9-OTUD5 mice after MI/R.

Group	AAV9-Control Sham	AAV9-OTUD5 Sham	AAV9-Control I/R	AAV9-OTUD5 I/R
n	5	5	5	5
Heart Rate (BPM)	422±19	425±4	445±4	454±5
LVESD (mm)	2.26±0.15	2.11±0.19	2.94±0.09*	2.35±0.10$^{\#}$
LVEDD (mm)	3.62±0.11	3.46±0.16	4.17±0.06*	3.32±0.12$^{\#\#\#}$
LVESV (μL)	17.89±3.11	15.51±3.52	33.52±2.55**	19.36±2.03$^{\#}$
LVEDV (μL)	51.36±0.82	50.28±5.66	68.88±1.76*	45.25±4.07$^{\#\#}$
LVAW,s (mm)	1.43±0.04	1.42±0.03	1.12±0.02***	1.22±0.05
LVAW,d (mm)	0.95±0.03	0.91±0.03	0.80±0.04*	0.83±0.03
LVPW,s (mm)	1.43±0.06	1.38±0.07	0.91±0.12**	1.21±0.05
LVPW,d (mm)	0.95±0.05	0.91±0.06	0.63±0.05**	0.60±0.02

Data are expressed as mean±SEM. $^{*}P$<0.05, $^{**}P$<0.01, $^{***}P$<0.001 *vs*. AAV9-Control Sham; $^{\#}P$<0.05, $^{\#\#}P$<0.01, $^{\#\#\#}P$<0.001 *vs*. AAV9-Control I/R.

Table S4. Baseline characteristics of control and myocardial ischemia patients.

Characteristic	Control（n=6）	Myocardial Ischemia（n=6）	*P-value*
Male	5(83.3%)	4(66.7%)	＞0.9999
Age	58.50±3.13	66±3.01	0.1148
**Previous history**			
Hypertension	4(66.7%)	3(50.0%)	＞0.9999
Diabetes mellitus	2(33.3%)	1(16.7%)	＞0.9999
Hyperlipidemia	2(33.3%)	1(16.7%)	＞0.9999
**Laboratory measurements**			
Cholesterol	4.05±0.34	4.36±0.56	0.6445
Triglyceride	1.40±0.32	0.98±0.13	0.2548
HDL	1.21±0.11	1.29±0.15	0.6548
LDL	2.22±0.23	2.48±0.40	0.5918
**Medication usage**			
Aspirin	3(50.0%)	4(66.7%)	＞0.9999
β-blocks	3(50.0%)	3(50.0%)	＞0.9999
ACE inhibitors or ARBs	2(33.7%)	3(50.0%)	＞0.9999

Table S5. List of the siRNA/shRNA sequences used in this study.

Genes（siRNA）	Sequence (5'-3')
si-GPX4-1	sense sequence: GCGUGUGCAUCGUCACCAATT
	antisense strand: UUGGUGACGAUGCACACGCTT
si-GPX4-2	sense sequence: CCGAGUGUGGUUUACGAAUTT
	antisense strand: AUUCGUAAACCACACUCGGTT
si-OTUD5-1	sense sequence: GGGACAAGAAAGGCUUCAUTT
	antisense strand: AUGAAGCCUUUCUUGUCCCTT
si-OTUD5-2	sense sequence: GUGGAGGUGUAUCAGUAUATT
	antisense strand: UAUACUGAUACACCUCCACTT
negative control (NC)	sense sequence: UUCUCCGAACGUGUCACGUTT
	antisense strand: ACGUGACACGUUCGGAGAATT
Genes (CRISPR-Cas9)	Sequence (5'-3')
sg-GPX4	sense sequence: TCGCAGCCAAGGACATCGAT
	antisense strand: ATCGATGTCCTTGGCTGCGA
sg-OTUD5	sense sequence: TTCGTTGGCCGGGTCGGCGT
	antisense strand: ACGCCGACCCGGCCAACGAA
sg-Con	sense sequence: CGCTTCCGCGGCCCGTTCAA
	antisense strand: TTGAACGGGCCGCGGAAGCG
